# Dibenzylideneacetone Induces Apoptosis in Cervical Cancer Cells through Ros-Mediated Mitochondrial Damage

**DOI:** 10.3390/antiox12020317

**Published:** 2023-01-30

**Authors:** Aline Pinto Zani, Caroline Pinto Zani, Zia Ud Din, Edson Rodrigues-Filho, Tânia Ueda-Nakamura, Francielle Pelegrin Garcia, Sueli de Oliveira Silva, Celso Vataru Nakamura

**Affiliations:** 1 Laboratory of Technological Innovation in the Development of Pharmaceuticals and Cosmetics, State University of Maringá, Maringá CEP 87020-900, PR, Brazil; 2 LaBioMMi, Department of Chemistry, Federal University of São Carlos, CP 676, São Carlos CEP 13565-905, SP, Brazil

**Keywords:** dibenzylideneacetone, apoptosis, oxidative stress, HeLa cell, SiHa cell

## Abstract

Cervical cancer is a health problem among women worldwide. Considering the limitations of prevention and antineoplastic chemotherapy against cervical cancer, research is needed to discover new, more effective, and safe antitumor agents. In the present study, we investigated the in vitro cytotoxicity of a new synthetic dibenzylideneacetone derived from 1,5-diaryl-3-oxo-1,4-pentadienyl (**A3K2A3**) against cervical cancer cells immortalized by HPV 16 (SiHa), and 18 (HeLa) by MTT assay. Furthermore, we performed spectrofluorimetry, flow cytometry, and Western blot analyzes to explore the inhibitory mechanism of **A3K2A3** in cervical cancer cells. **A3K2A3** showed cytotoxic activity against both cell lines. Mitochondrial depolarization and reduction in intracellular ATP levels were observed, which may be dependent on the redox imbalance between increased ROS and reduced levels of the antioxidant defense. In addition, damage to the cell membrane and DNA, and effective blocking of cell division in the G2/M phase were detected, which possibly led to the induction of apoptosis. This result was further confirmed by the upregulation of apoptosis-related proteins Bax, cytochrome C, and caspases 9 and 3. Our results provided the first evidence that **A3K2A3** contributes to the suppression of cervical cancer in vitro, showing promise as a possible alternative for the treatment of this cancer.

## 1. Introduction

Cervical cancer is the fourth most common cancer among women worldwide, with an estimated 604,000 new cases and 342,000 deaths in 2020. More than 95% of cervical cancer cases are infection-related with high-risk for human papillomavirus (HPV) [1]. Among the high-risk HPVs, HPV16 and HPV18 are responsible for almost 70% of these cases [2,3].

In the last decade, screening for HPV lesions through HPV and Pap smear tests, and prevention through HPV vaccinations have been recognized as the most effective interventions to control the mortality rate associated with this disease [3,4]. Despite these screening and prevention programs, the incidence of cervical cancer is still one of the leading causes of cancer-related death in women [5]. This scenario may be due to the fact that screening tests are not widely available in many underdeveloped countries, a situation related to poverty and the lack of resources and infrastructure [6]. Further, vaccination is limited to young people to ensure effectiveness [7]. Even with recent advances, the clinical treatments available for cervical cancer, including radiotherapy, chemotherapy, and surgery, are still not wholly adequate due to the frequent occurrence of adverse effects, among other limitations, such as systemic toxicity and chemoresistance [8,9].

The need to identify new, more effective, and safer antitumor agents becomes clearly justifiable. In general, natural products have demonstrated advantages due to their availability and biological potential and, in recent decades, several natural products have been studied as potential anticancer drugs, either in their unmodified (natural) or modified (semi-synthetic) forms [10,11]. Chalcone and curcumin derivatives have shown relevant cytotoxic activity, in addition to the regulation of multiple signaling pathways in cancer cell lines [11,12,13,14,15,16].

In this sense, compounds containing these derivatives, or similar groups, in their structure are promising, leading to growing interest in the research to obtain antitumor agents. Based on structural similarities with chalcones and curcumins, dibenzylideneacetones (DBAs) were synthesized and characterized by Ud Din [17]. DBAs are a class of synthetic compounds that have an acyclic dienone attached to aryl groups at both β positions [17].

Studies have demonstrated the therapeutic properties of DBAs, including as anticancer agents, by inhibiting STAT3 signaling, an oncogenic transcription factor that controls the expression of genes associated with oncogenesis and malignant progression in preclinical models of hepatocellular carcinoma (HCC) and multiple myeloma (MM) [18]. DBA further exhibited antitumor activity in mice xenografted with human mucoepidermoid cells (ECM) by regulating Sp1, and Bim and t-Bid proteins without causing toxicity [19], and further inhibited cell growth and induced apoptosis in several human oral cancer cell lines [20]. Furthermore, DBAs have been observed increasing apoptosis by regulating cell survival and pro-apoptotic proteins through the activation of reactive oxygen species (ROS) and CCAAT/Enhancer Binding Protein Homologous Protein (CHOP) in colon cancer cells [21]. Our research group reported that DBA (1E,4E)-2-methyl-1,5-bis(4- nitrophenyl)penta-1,4-dien-3-one (**A3K2A3**) shows trypanocidal activity against protozoa of the genera *Leishmania* and *Trypanosoma cruzi*, and is capable of causing oxidative damage and induction of parasite cell death via necrosis and/or apoptosis [22,23,24]. There is, however, a lack of studies on cervical cancer.

Considering the challenges of treating cervical cancer, and the diverse therapeutic properties already described for DBAs and their derivatives, this study was conducted with the compound **A3K2A3**, a new synthetic DBA, against cervical cancer cell lines (HeLa and SiHa), seeking to develop a new strategy capable of interfering with its proliferation. We further investigated the effects of this compound on different pathways (mitochondrial, oxidative, apoptotic, and necrotic) in order to understand the possible mechanism of action involved in the cell death of these cell lines.

The induction of apoptosis in cancer cells is considered the main way to inhibit the proliferation of tumor cells, providing an important indication for the development of potential anticancer drugs [25,26]. Among these approaches, the modulation of ROS may represent another strategy for killing cancer cells [27] through the imbalance between antioxidant defense mechanisms and ROS production, which generates oxidative stress and can induce cell death, through apoptosis [28,29]. Another feature of programmed cell death (PCD) is the considerable cell shrinkage or decrease in cell volume that is a necessary event during the apoptosis process. In addition, the literature reports that resistance to apoptosis can be developed when cell volume reduction is avoided [30].

Our results provide the first evidence that DBA **A3K2A3** contributes to the suppression of cervical cancer in vitro, as demonstrated by the selective cytotoxic effect of **A3K2A3** on cancer cells and by its mechanism of action through the increased production of reactive species of oxygen and reduced levels of antioxidant defenses, which possibly contributed to apoptotic cell death.

## 2. Materials and Methods

### 2.1. Reagents

Secondary mouse IgGκ BP-HRP antibody (sc-516102), mouse anti-Bax (sc-20067), mouse anti-Bcl2 (sc-7382), mouse anti-caspase 9 (sc-56076), mouse anti-caspase 3 (sc-56053), mouse anti-matrix metalloproteinase-9 (MMP-9) (sc-93859), mouse anti-cytochrome C (sc-13561), and mouse anti-β-actin (sc-69879) were purchased from Santa Cruz Biotechnology (SCBT) (Santa Cruz, CA, USA). Bradford’s reagent was obtained from Bio-Rad. CellTiter-Glo was acquired from Promega. Annexin V fit-C, Agarose, Camptothecin (CPT), DNase-free RNase, dichlorodihydrofluorescein diacetate (H_2_DCFDA), Hoechst-33342 (2’-[4-ethoxyphenyl]-5-[4-methyl-1-piperazinyl]-2,5’-bi-1H-benzimidazole trihydrate), 3-(4,5-dimethylthiazol-2-yl)-2,5-diphenyltetrazolium bromide (MTT), diphenyl-1-pyrenylphosphine (DPPP), Proteinase K, and SYBR^®^ Safe DNA dye were purchased from Invitrogen (Eugene, Oregon, USA). Dulbecco’s Modified Eagle Medium (DMEM) and Fetal Bovine Serum (SFB) were purchased from Gibco (Grand Island, NE, USA). Bovine serum albumin (BSA), Carbonylcyanide m-chlorophenylhydrazone (CCCP), Dimethylsulfoxide (DMSO), glycine, glutathione reduced (GSH), acridine orange (AO), tetramethylrhodamine ethyl ester (TMRE), digitonin, propidium iodide (IP), o- phthalaldehyde (OPA), hydrogen peroxide (H_2_O_2_), potassium cyanide (KCN), and Rhodamine 123 (Rh123) were purchased from Sigma Aldrich (St Louis, MO, USA).

### 2.2. Compound

**Figure 1:**
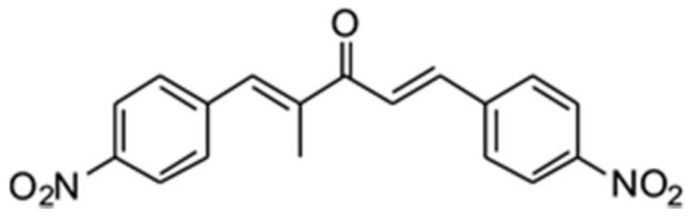
Chemical structure of (1E, 4E)-2-methyl-1,5-bis(4-nitrophenyl) penta-1,4-dien-3-one (**A3K2A3**).

The compound (1E, 4E)-2-methyl-1,5-bis(4-nitrophenyl) penta-1,4-dien-3-one (**A3K2A3**), is a synthetic dibenzylideneacetone with a molecular weight of 338.3 g/mol and was synthesized as previously described by Ud Din [17]. Its molecular structure is shown in Figure 1. **A3K2A3** stock solutions were prepared aseptically in DMSO and diluted in a culture medium so that the DMSO concentration did not exceed 1% in the experiments. The concentrations of **A3K2A3** that were used in all assays were based on concentrations corresponding to inhibitory concentration for 50% of the cells (IC_50_) (18.9 and 17.4 µM for HeLa and SiHa, respectively) and 2xIC_50_ (37.8 and 34.8 µM for HeLa and SiHa, respectively) obtained after 48 h of treatment. The first assays were carried out using the treatment times of 24 and 48 h in order to investigate the initial changes in the cells, and at 48 h to confirm these observed results. The other assays were performed only after 48 h of treatment when there was already an indication as to the type of death induced in the cells.

### 2.3. Cell Lines and Culture Conditions

Cervical cancer cell lines HeLa (HPV 18-positive) and SiHa (HPV 16-positive) as well as immortalized human keratinocytes (HaCaT) were provided by Dr. Luisa L. Villa (ICESP, Faculty of Medicine, University of São Paulo/Brazil) and Dr. Silvya S. Maria-Engler (Faculty of Pharmaceutical Sciences, University of São Paulo/Brazil). All cell lines were maintained in a culture flask in DMEM supplemented with 2 mM L-glutamine, 10% of FBS, and 0.5 U/mL penicillin/streptomycin at 37 °C in an atmosphere of 5% CO_2_.

### 2.4. Cytotoxic Activity

The evaluation of **A3K2A3** cytotoxicity was performed with the cell lines HeLa, SiHa, and HaCaT using the MTT colorimetric assay. The MTT assay is based on the ability of viable cells via mitochondrial dehydrogenase to reduce MTT, which is a salt of tretrazolium formazan crystal violet [10]. For this, each cell line was resuspended at a concentration of 2.5 × 10^5^ cells/mL in DMEM medium. Then, 100 μL/well was added to 96-well plates, and the plates were incubated at 37 °C with 5% CO_2_ for 24 h. After reaching at least 80% confluence of the monolayer, the compound **A3K2A3** was added at concentrations of 1, 10, 50, and 100 µM, and incubated for 48 h at 37 °C with 5% CO_2_. Afterward, the wells were washed with 100 µL of phosphate-buffered saline (PBS), and 50 µL of MTT solution (2 mg/mL) was added, followed by incubation for 4 h under the same conditions described above (protected from light). Subsequently, 150 µL of DMSO was added and the reading was performed in a microplate reader (Bio-Tek^®^, Power Wave XS, Winooski, VT, USA) at 570 nm. The analysis was performed using non-linear regression in which the % cell viability was calculated according to the equation:

% Cell Viability = (Experimental Absorbance/Average Control Absorbance) × 100 (1)

The IC_50_ was determined as the concentration capable of reducing 50% of the optical density of the treated cells compared to the untreated cells.

### 2.5. Assessment of the Morphology of Cervical Cancer Cells

#### 2.5.1. Phase Contrast Microscopy

HeLa, SiHa, and HaCaT cells were plated at the concentration of 2.5 × 10^5^ cells/mL with DMEM medium in a 24-well plate and were incubated at 37 °C with 5% CO_2_ for 24 h. After reaching at least 80% confluence of the monolayer, treatment was performed with **A3K2A3** (IC_50_ and 2xIC_50_ according to cell line), and HaCaT cells were treated with concentrations higher than the IC_50_ and 2xIC_50_ of cancer cells (19 and 38 µM). The cells were then incubated at 37 °C with 5% CO_2_. Cell morphology was observed after 24 and 48 h of treatment, and images were taken using an inverted phase contrast microscope (20×; Olympus CKX41 coupled with the SC30 camera).

#### 2.5.2. Scanning Electron Microscopy (SEM)

HeLa, SiHa, and HaCaT cells were plated at a density of 2.5 × 10^5^ cells/mL in a 24-well culture plate containing a sterile round coverslip, followed by incubation at 37 °C with 5% CO_2_ for 24 h. The monolayer was then treated with **A3K2A3** (IC_50_ and 2xIC_50_ according to cell line), and HaCaT cells were treated with concentrations higher than the IC_50_ and 2xIC_50_ of cancer cells (19 and 38 µM). Plates were incubated at 37 °C with 5% CO_2_ for 24 and 48 h. After this period, the wells were washed once in PBS buffer solution and the cells were fixed with 2.5% glutaraldehyde in 0.1 M sodium cacodylate buffer (pH 7.4) for 90 min at 8 °C. They were then dehydrated in an increasing series of ethanol (30, 50, 70, 90, 95, and 100%), critical point dried, metalized, and finally analyzed in the scanning electron microscope FEI Quanta 250.

### 2.6. Determination of Cell Volume

Cell volume was analyzed according to the methodology adapted from Lazarin-Bidóia [23]. HeLa and SiHa cells were plated at a density of 2.5 × 10^5^ cells/mL in DMEM medium by adding 2000 μL/well to a 6-well plate. It was then incubated at 37 °C with 5% CO_2_ for 24 h. The cells were then treated with **A3K2A3** (IC_50_ and 2xIC_50_ according to cell line) for 24 and 48 h. The positive control (PC) was carried out with CPT 30 μM. Cells were then washed twice in PBS and resuspended in the same buffer. Then, the cell volume was analyzed by a FACSCalibur flow cytometer (Becton–Dickinson, Rutherford, NJ, USA). Histograms and analyses were performed using CellQuest software (Joseph Trotter, Scripps Research Institute, La Jolla, CA, USA). A total of 10,000 events were acquired.

### 2.7. ROS Production

Total ROS production was evaluated based on the increase in fluorescence caused by the conversion of non-fluorescent dye to highly fluorescent 2′,7′-dichlorodihydrofluorescein diacetate [31]. HeLa and SiHa cells were plated at a density of 2.5 × 10^5^ cells/mL in a 24-well plate, and it was incubated at 37 °C with 5% CO_2_ for 24 h. The cells were then treated with **A3K2A3** (IC_50_ and 2xIC_50_ according to cell line) for 24 and 48 h. The PC was carried out with H_2_O_2_ (200 μM). Cells were then washed with PBS, detached by trypsinization, resuspended in PBS, and labeled with H_2_DCFDA (10 μM) for 30 min in the dark at room temperature. The fluorescence intensity was quantified using a fluorescence microplate reader (Victor^®^ X3, Perkin Elmer, Waltham, MA, USA) at excitation and emission wavelengths of 488 and 530 nm, respectively. The fluorescence intensity was then normalized to the number of cells [10]. For analysis under fluorescence microscopy (Olympus^®^, BX51, Shinjuku, Tokyo), the same labeling procedure was performed and the images were captured with an Olympus^®^ UC30 camera.

### 2.8. GSH Levels

GSH levels were determined using the methodology described by Daré [32]. Briefly, HeLa and SiHa cells were plated (2.5 × 10^5^ cells/mL) in a 24-well plate, followed by incubation at 37 °C with 5% CO_2_ for 24 h. The cells were then treated with **A3K2A3** (IC_50_ and 2xIC_50_ according to cell line) for 24 and 48 h. The PC was carried out with H_2_O_2_ (200 μM). Subsequently, cell lysates were prepared by scraping cells in ice-cold lysis buffer [10 mM Tris-HCl (pH 7.4) and 1% triton X100], followed by sonication for 60 s (30% amplitude, 5 s on and 5 s off) and centrifugation at 10,000 g/10 min/4 °C. Supernatants were collected, protein quantification was performed, and cell lysates were stored at −80 °C until assay. One hundred and Eighty μL of sodium phosphate buffer (0.1 M, 5 mM EDTA, pH 8.0), 10μL of cell lysate, 10 μL of OPT, and 1 mg/mL in methanol were added to a 96-well black plate. The fluorescence was determined after 15 min of incubation at room temperature at 350/420nm excitation/emission wavelengths in a spectrofluorimeter (Victor^®^ X3, Perkin Elmer). A calibration curve with GSH (1.953 to 1000 μg/mL) was used to calculate the results, which were expressed as μg GSH/mg of protein.

### 2.9. Assessment of Mitochondrial Membrane Potential (Δψm)

#### 2.9.1. TMRE Labeling

The evaluation of ΔΨm was first performed using TMRE, a positively charged compound, which accumulates active mitochondria due to the presence of negative charges [33]. Five hundred μL/well of HeLa and SiHa cells (2.5 × 10^5^ cells/mL) were added to a 24-well plate and incubated at 37 °C with 5% CO_2_ for 24 h. After that, the cells were treated with **A3K2A3** (IC_50_ and 2xIC_50_ according to cell line) for 24 and 48 h. The PC was carried out with CCCP (100 μM). The cells were washed with PBS, detached by trypsinization, resuspended in PBS, and labeled with TMRE (25 nM) for 30 min in the dark at 37 °C under a 5% CO_2_ atmosphere. The fluorescence intensity was quantified in a fluorescence microplate reader (Victor^®^ X3, Perkin Elmer) at excitation and emission wavelengths of 540 and 595 nm, respectively. The fluorescence intensity was normalized to the number of cells [10].

#### 2.9.2. Marking with Rh123

The Δψm was further analyzed according to the methodology adapted from Lazarin-Bidóia [23] using Rh123, a fluorescent probe that accumulates in intact or active mitochondria [34]. Briefly, HeLa and SiHa cells were plated (2.5 × 10^5^ cells/mL) in a 6-well plate, which was incubated at 37 °C with 5% CO_2_ for 24 h. Cells were treated with **A3K2A3** (IC_50_ and 2xIC_50_ according to cell line) for 24 and 48 h. The PC was carried out with CCCP (100 μM). After treatment, cells were washed with PBS buffer and labeled with 5 µg/mL Rh123 solution for 15 min at 37 °C. The cells were then washed and resuspended in PBS for analysis on a FACSCalibur flow cytometer (Becton-Dickinson, Rutherford, NJ, USA) equipped with CellQuest software (Joseph Trotter, Scripps Research Institute, La Jolla, CA, USA). A total of 10,000 events were acquired in the region previously established with the cells.

### 2.10. Intracellular Adenosine Triphosphate (ATP) Level Determination

ATP levels were measured using an ATP Cell Titer-Glo^®^ Assay Kit, following the manufacturer’s instructions. HeLa and SiHa cells were plated (2.5 × 105 cells/mL) in a 24-well plate, which was incubated at 37 °C with 5% CO_2_ for 24 h. Cells were then treated with **A3K2A3** (IC_50_ and 2xIC_50_ according to cell line) for 24 and 48 h. The PC was carried out with KCN (1000 μM). The cells were washed with PBS, detached by trypsinization, resuspended in PBS, and marked with Cell Titer-Glo reagent for 10 min at room temperature. The luminescence intensity was quantified using a fluorescence microplate reader (Victor^®^ X3, Perkin Elmer). The fluorescence intensity was normalized to the number of cells [10].

### 2.11. Cell Membrane Assessment

#### 2.11.1. Lipid Peroxidation (LPO)

LPO was determined based on the amount of DPPP. DPPP is essentially non-fluorescent until it reacts with lipid peroxides and hydroperoxides and is oxidized into the fluorescent substance phosphine oxide (DPPP-O) [35]. HeLa and SiHa cells were plated (2.5 × 10^5^ cells/mL) in a 24-well plate, which was incubated at 37 °C with 5% CO_2_ for 24 h. The cells were then treated with **A3K2A3** (IC_50_ and 2xIC_50_ according to cell line) for 24 and 48 h. The PC was carried out with H_2_O_2_ (200 μM). The cells were then washed with PBS, detached by trypsinization, resuspended in PBS, and labeled with DPPP (50 μm) for 15 min in the dark at room temperature. The fluorescence intensity was quantified in a fluorescence microplate reader (Victor^®^ X3, Perkin Elmer) at excitation and emission wavelengths of 351 and 460 nm, respectively. The fluorescence intensity was normalized to the number of cells [10].

#### 2.11.2. Cell Membrane Integrity

The assessment of cell membrane integrity was performed using PI. PI Dye is a deoxyribonucleic acid (DNA) intercalating agent, not permeable to intact plasma membranes [36]. For this, HeLa and SiHa cells were plated (2.5 × 10^5^ cells/mL) in a 24-well plate and then incubated at 37 °C with 5% CO_2_ for 24 h. After that, the cells were treated with **A3K2A3** (IC_50_ and 2xIC_50_ according to cell line) for 24 and 48 h. The PC was carried out with digitonin (80 μM). The cells were washed with PBS, detached by trypsinization, resuspended in PBS, and labeled with PI (4 μg/mL) for 5 min in the dark at room temperature. The fluorescence intensity was quantified in a fluorescence microplate reader (Victor^®^ X3, Perkin Elmer) at excitation and emission wavelengths of 480 and 580 nm, respectively. The fluorescence intensity was normalized to the number of cells [10].

### 2.12. DNA Damage Evaluation

#### 2.12.1. Cell Cycle

HeLa and SiHa cells were plated at a density of 2.5 × 10^5^ cells/mL in a 6-well plate (2000 μL/well) and incubated at 37 °C with 5% CO_2_ for 24 h. The cells were then washed with PBS and treated with **A3K2A3** (IC_50_ and 2xIC_50_ according to cell line) for 24 and 48 h. Cells were then fixed in 70% (*v*/*v*) methanol/PBS at 4 °C for 1 h. Afterward, they were washed and incubated in PBS containing 10 µg/mL of PI and 20 µg/mL of RNAse at 37 º C for 45 min. Data acquisition was performed immediately after labeling, using a FACSCalibur flow cytometer (Becton-Dickinson, Rutherford, NJ, USA) and the data were analyzed using CellQuest software (Joseph Trotter, Scripps Research Institute, La Jolla, CA, USA) [23]. A total of 10,000 events were collected per sample and the data was analyzed using Modfit LT 5.0 software.

#### 2.12.2. Hoechst 33342 Staining

The nuclear condensation was examined using the cell-permeable DNA dye Hoechst 33342. For this, HeLa and SiHa cells were plated (2.5 × 10^5^ cells/mL) in a 24-well culture plate (500 μL/well) containing a sterile round coverslip and incubated at 37 °C with 5% CO_2_ for 24 h. The cells were then treated with **A3K2A3** (IC_50_ and 2xIC_50_ according to cell line) for 48 h. The PC was carried out with CPT (10 μM). The cells were washed with PBS, labeled with 10 μg/mL Hoechst 33342, incubated for 20 min in the dark at 37 °C, and then washed again twice with PBS. They were then visualized on a fluorescence microscope (Olympus^®^, BX51) and the images were captured with an Olympus^®^ UC30 camera. The identification criteria were living cells with homogeneously stained nuclei (light blue), while cells strongly stained with an intense and bright blue color were indicative of apoptosis due to chromatin condensation. The images were analyzed using ImageJ software [32].

#### 2.12.3. Agarose Gel Electrophoresis

According to the methodology adapted from Daré [32], DNA fragmentation was evaluated by the agarose gel electrophoresis method. Briefly, HeLa and SiHa cells were resuspended at a density of 2.5 × 10^5^ cells/mL and 2000 µL/well were added to a 6-well culture plate, which was incubated for 24 h at 37 °C with 5% CO_2_. The cells were then treated with **A3K2A3** (IC_50_ and 2xIC_50_ according to cell line) for 48 h. The PC was carried out with CPT (30 μM). After the treatment, the cells were lysed in a solution containing Tris-HCl (10 mM; pH 8), EDTA (1 mM), NaCl (100 mM), SDS (0.5%) and proteinase k (20 mg/mL) at 65 °C for 15 min. Then, RNAse (1 mg/mL) was added and the solutions were incubated at 37 °C for 15 min. DNA was purified using phenol: chloroform: isoamyl alcohol (25:24:1; *v*/*v*). DNA electrophoresis was performed on a 1% agarose gel in Tris/boric acid/EDTA at 90 V for 1 h, labeled with SYBR safe (Invitrogen). The molecular weight marker used was the 100 bp DNA Ladder. Images of DNA fragmentation were obtained using the GelDoc^®^ XR + Imaging System equipment (Bio-Rad Laboratories Inc., Hercules, CA, USA).

### 2.13. Cell Death Assessment

#### 2.13.1. Detection of Phosphatidylserine Exposure

The Annexin-V FITC kit allows the detection of apoptosis by labeling phosphatidylserine molecules that translocate to the outside of the cell membrane. By also associating PI, it is possible to measure cells with altered membrane permeability. Labeling with Annexin or double labeling (Annexin-V/PI) enables the interpretation of the cells as being in early and late apoptosis, respectively, and labeling only with PI indicates necrosis [37,38]. HeLa and SiHa cells were plated (2.5 × 10^5^ cells/mL) in a 6-well culture plate (2000 µL/well) and incubated for 24 h at 37 °C with 5% CO_2_. The cells were then washed with PBS and treated with **A3K2A3** (IC_50_ and 2xIC_50_ according to cell line) for 24 and 48 h. Cells were then washed and resuspended in 100 µL binding buffer (140 mM NaCl, 5 mM CaCl_2_, 10 mM HEPES-Na, pH 7.4), followed by the addition of 5 µL Annexin-V FITC, and incubation for 15 min at room temperature. Afterward, 400 µL of binding buffer and 50 µL of PI were added. Data acquisition and analysis were performed using the FACSCalibur flow cytometer (Becton-Dickinson, Rutherford, NJ, USA) equipped with CellQuest software (Joseph Trotter, Scripps Research Institute, La Jolla, CA, USA). A total of 10,000 events were collected [39].

#### 2.13.2. Dual Staining of AO and PI

Analysis of cell death by double staining with AO and PI was evaluated as described by Kaplum [40]. HeLa and SiHa cells were plated (2.5 × 10^5^ cells/mL) in a 24-well culture plate (500 μL/well) containing a sterile round coverslip and incubated at 37 °C with 5% CO_2_ for 24 h. They were then treated with **A3K2A3** (IC_50_ and 2xIC_50_ according to cell line) for 48 h. The PC was carried out with CPT (10 μM). Cells were then washed with PBS and labeled with AO (10 μg/mL) and PI (4 μg/mL) for 15 min in the dark. Fluorescence was analyzed using an Olympus BX51 fluorescence microscope and images were captured using a UC30 camera within 30 min. The identification criteria were: viable cells had a green nucleus with intact structures, cells in early apoptosis had a bright green nucleus indicating chromatin condensation, and cells in late apoptosis showed AO/PI double labeling as a result of changes in the cell membrane and formation of apoptotic bodies with dense orange areas of condensed chromatin and bright reddish-orange nuclei indicating necrosis. The number of viable cells in early apoptosis, late apoptosis, and in necrosis was determined by counting 200 cells in triplicate.

#### 2.13.3. Western Blotting

The analysis of protein expression was performed according to the methodology adapted from Daré [32]. HeLa and SiHa cells were plated at a density of 2.5 × 10^5^ cells/mL (2000 µL/well) in a 6-well culture plate and incubated for 24 h at 37 °C with 5% CO_2_. The cells were then washed with PBS and treated with **A3K2A3** (IC_50_ and 2xIC_50_ according to cell line) for 48 h. The cells were detached using a cell scraper, and floating and scraped spontaneous cells were centrifuged at 1000 g/4 °C/10 min. The obtained pellet was washed twice with PBS and resuspended in ice-cold lysis buffer [150 mM sodium chloride, 5 mM EDTA, 50 mM Tris-HCl (pH 8.0), 1% Triton X-100, 5% SDS, and 1% protease inhibitor cocktail. Cell lysates were sonicated for 2 min (4C15, Branson Ultrasonics^®^, Fairfield County, CT, USA; 60% amplitude, 20 s on and 20 s off) and centrifuged at 10,000 g/20 min/4 °C. The amount of protein was estimated with Bradford’s reagent. Then, equal amounts of protein were collected and heated at 100 °C for 5 min in the loading buffer [5% mercaptoethanol, 5% bromophenol blue, 75 mM Tris-HCl (pH 6.8), 2% SDS, and 10% glycerol]. The proteins were subjected to 12% SDS-polyacrylamide gel electrophoresis, then transferred to a nitrocellulose membrane (GE-Healthcare, Solingen, Germany) in the transfer buffer (25 mM Tris base, 192 mM glycine, 20% methanol, and 0.01%). Membranes were blocked with 5% albumin in TBS-T buffer [25 mM Tris-HCl (pH 7.4), 150 mM NaCl and 0.1% tween 20] for 1 h at room temperature and incubated overnight at 4 °C with the primary antibodies (MMP-9, Bax, Bcl-2, Cytochrome C, caspase 9 and 3) diluted 1:500, and β-actin diluted 1:10000 (SCBT, CA, USA) in TBS-T buffer with 3% albumin. Membranes were washed with TBS-T buffer and incubated with the secondary mouse IgGκ BP-HRP antibody, diluted 1:5000 for 1 h at room temperature. The antigen-antibody complex was detected by chemiluminescence with the ECL detection reagent (SCBT, CA, USA) and analyzed with the ChemiDoc^®^ XRS + Imaging System (Bio-Rad Lab. Inc., CA, USA).

### 2.14. Clonogenic Assay

HeLa and SiHa cells were plated at a density of 2.5 × 10^5^ cells/mL in a 6-well culture plate (2000 µL/well) and incubated for 24 h at 37 °C with 5% CO_2_. The treatment was then performed with **A3K2A3** (IC_50_ and 2xIC_50_ according to cell line) for 24 and 48 h at 37 °C under a 5% CO_2_ atmosphere. The medium was then removed and the cells were again incubated with pure culture medium for ten days. The culture medium was replaced every 72 h. The colonies formed were washed with PBS, fixed in ice-cold methanol (100%) for 10 min, washed again with PBS, and stained with 5% Giemsa for 40 min. A collection of cells was considered a colony when there were more than 50 cells [41].

### 2.15. Wound Healing Assay

The wound healing assay evaluates the ability of cell migration in vitro and was performed as described by Kaplum [40]. Briefly, HeLa and SiHa cells were plated (2.5 × 10^5^ cells/mL) in a 24-well cell culture plate for 24 h at 37 °C under a 5% CO_2_ atmosphere. The cells were then incubated with 0.5% heat-inactivated FBS for 6 h. After that, the cells were scratched with a sterile 200 µL pipette tip, washed with PBS, and treated with **A3K2A3** (IC_50_ and 2xIC_50_ according to cell line) for 24 and 48 h at 37 °C under a 5% CO_2_ atmosphere. Cell migration was observed under a phase-contrast inverted microscope (Olympus CKX4; SC30 camera; 4× magnification). The percentage of cell growth in the wound region was calculated by ImageJ software to determine the cell migration capacity.

### 2.16. Statistical Analysis

Statistical analyzes were performed using GraphPad Prism software version 8.00 (San Diego, CA, USA). The data shown in the table and graphs express the mean ± standard deviation of at least three independent experiments. Data were analyzed using ANOVA tests (one-way or two-way), followed by Tukey’s post-test, considering *p* < 0.05 as significant.

## 3. Results

### 3.1. Cytotoxic Activity of A3K2A3 in Cervical Cancer Cell Lines

**Figure 2:**
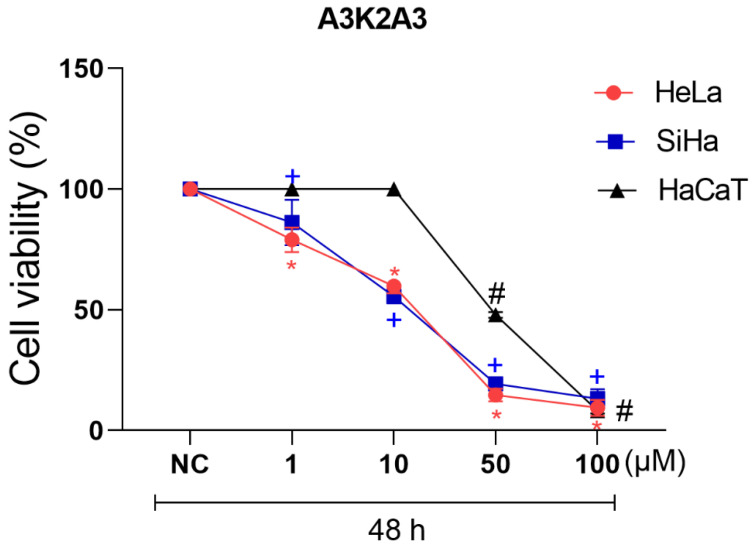
Cytotoxic activity in cervical cancer cells and HaCaT. Cells were treated with **A3K2A3** (1–100 µM) for 48 h, and cell viability was assessed using the MTT assay. Data represent the mean ± SD of three independent experiments that were performed in triplicate. HeLa; SiHa; HaCaT * *p* < 0.05 compared to 48 h NC of HeLa cells, + *p* < 0.05 compared to 48 h NC of SiHa cells, # *p* < 0.05 compared to 48 h negative control (NC) of HaCaT cells; (two-way ANOVA followed by Tukey’s post-test).

Compound **A3K2A3** showed dose-dependent cytotoxic activity in both HeLa and SiHa cervical cancer cell lines (Figure 2). The IC_50_ determined after 48 h of treatment is presented in Table 1. Furthermore, in order to determine the selectivity index (SI), cytotoxicity on HaCaT cells was performed and the IC_50_ was determined after 48 h of treatment (Table 1). Thus, the cytotoxicity of the **A3K2A3** compound was greater in HeLa and SiHa cells compared to non-tumor cells (HaCaT), presenting a SI > 2 for HeLa and SiHa (Table 1).

### 3.2. Effects of A3K2A3 on Cell Morphology

#### A3K2A3 Induces Changes in the Morphology of Cervical Cancer Cells Visualized by Optical and SEM

**Figure 3:**
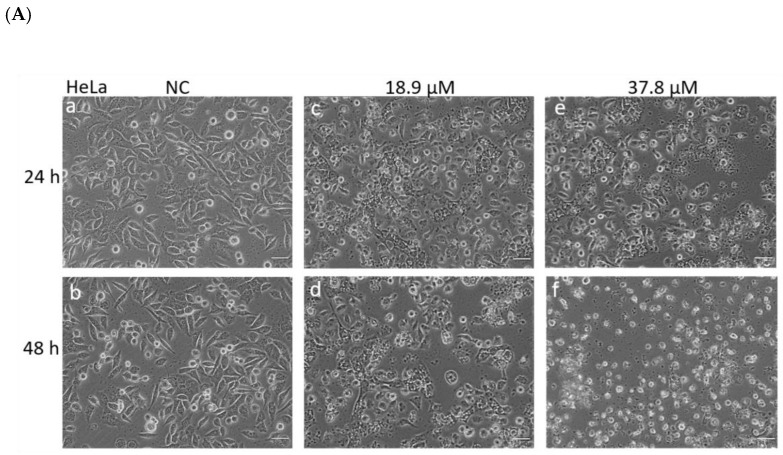

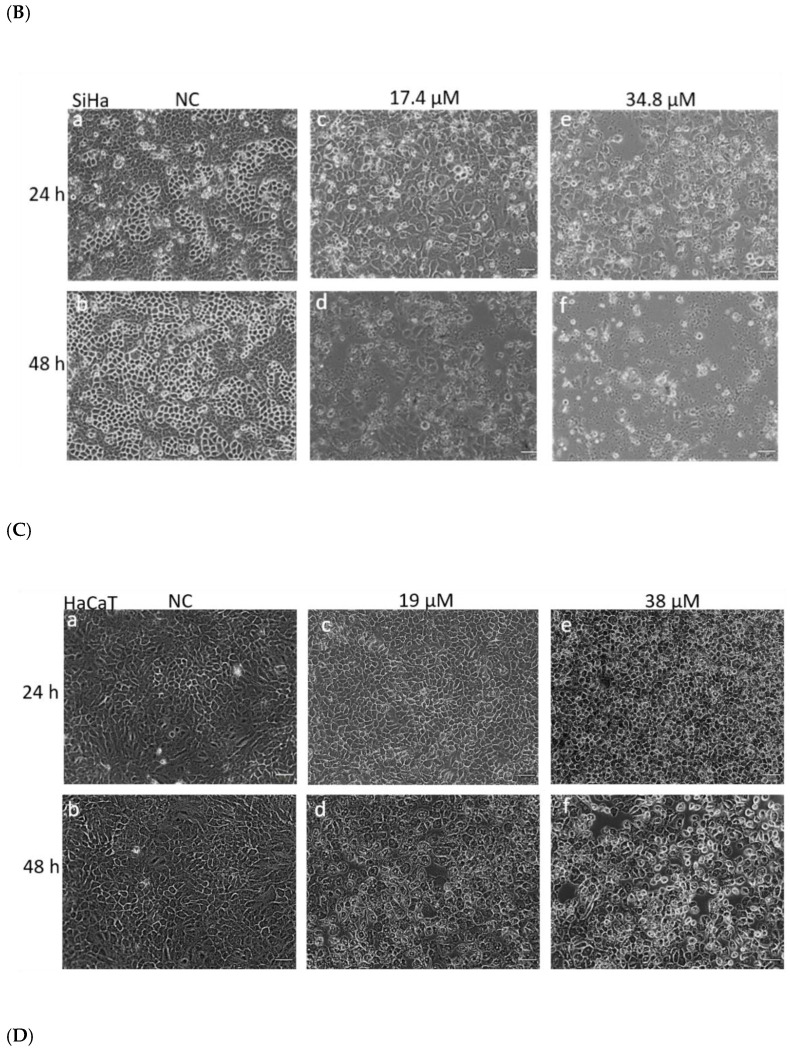

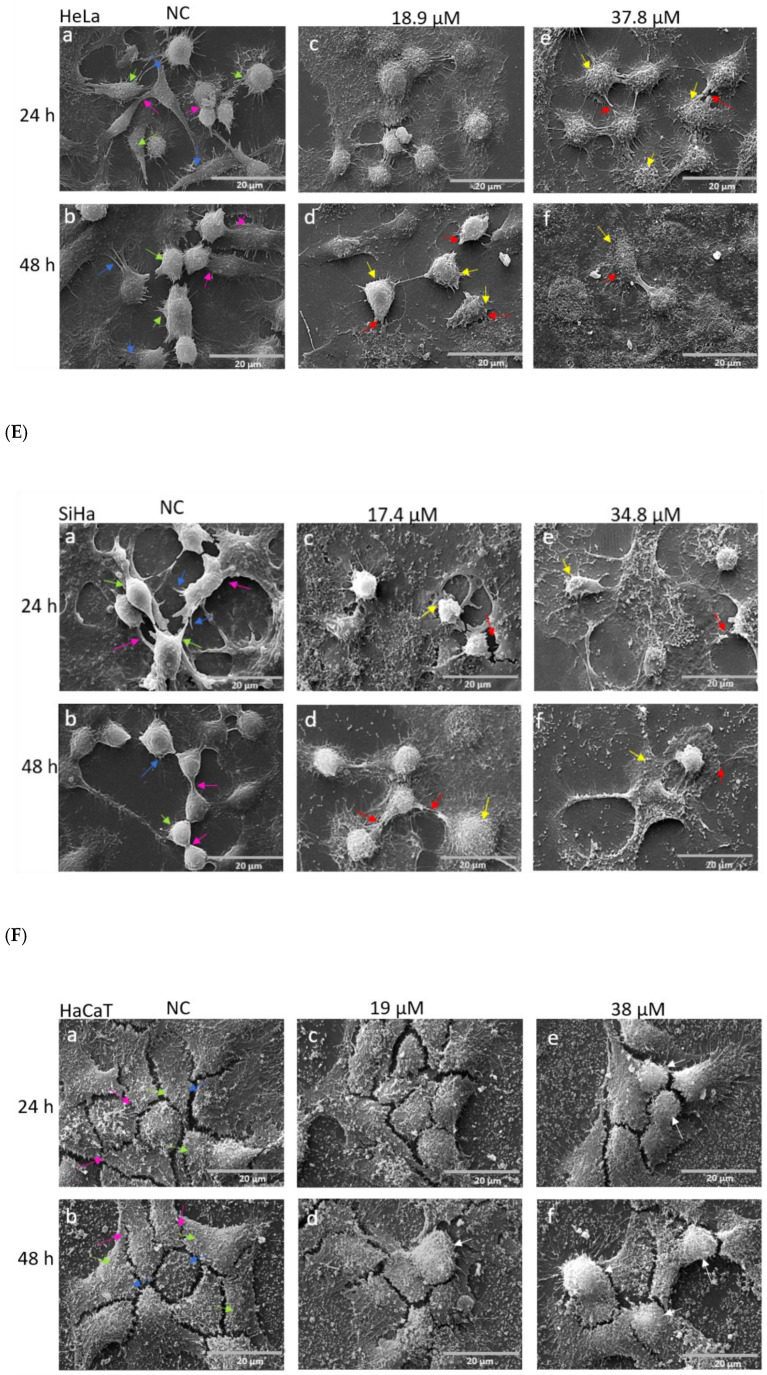
Analysis of cell morphology in cervical cancer cell lines and immortalized human keratinocytes. (**A**) HeLa and (**B**) SiHa: (**a**,**b**) NC at 24 and 48 h, respectively. (**c**,**e**) Cells treated with A3K2A3 (IC_50_ or 2xIC_50_) for 24 h. (**d**,**f**) Cells treated with A3K2A3 (IC_50_ or 2xIC_50_) for 48 h, (**C**) HaCaT:(**a**,**b**) NC at 24 and 48 h, respectively. (**c**,**e**) Cells treated with A3K2A3 (19 or 39 µM) for 24 h. (**d**,**f**) Cells treated with A3K2A3 (19 or 39 µM) for 48 h, images were performed using an Olympus CKX41 inverted phase contrast microscope coupled with an SC30 camera (20× magnification; scale bar: 50 μm). (**D**) HeLa and (**E**) SiHa: (**a**,**b**) NC at 24 and 48 h, respectively. (**c**,**e**) Cells treated with A3K2A3 (IC_50_ or 2xIC_50_) for 24 h. (**d**,**f**) Cells treated with A3K2A3 (IC_50_ or 2xIC_50_) for 48 h, (**F**) HaCaT:(**a**,**b**) NC at 24 and 48 h, respectively. (**c**,**e**) Cells treated with A3K2A3 (19 or 39 µM) for 24 h. (**d**,**f**) Cells treated with A3K2A3 (19 or 39 µM) for 48 h. Images were performed in SEM Quanta 250 (FEI) Magnification: 5000× 6500×.

The morphology of HeLa, SiHa, and HaCaT cells was first evaluated under an inverted phase contrast microscope, as shown in Figure 3 (A, B, and C, respectively). After treatment for 24 h with IC_50_ and 2xIC_50_ for HeLa and SiHa, changes in shape and a decrease in cell volume were observed, with these changes being more evident after 48 h of treatment and when using the 2xIC_50_ concentration, when it was possible to observe increased irregularity of shape, cell detachment and loss of intercellular contacts (Figure 3A,B; images c–f). When HaCaT cells were treated with 19 and 39 µM (higher concentrations than the IC_50_ and 2xIC_50_ for cancer cells) did not show many morphological changes in relation to the NC. Only a rounding of these cells was observed, which intensified in the treatment for 48 h, and there was no significant reduction in the number of cells in relation to the NC.

Aiming to confirm these previous data, morphological analysis was also performed by scanning electron microscopy (Figure 3D–F). The NC cells (Figure 3D,E; images a,b) showed regular morphology with an intact membrane, multiple membrane connections, and an elongated spindle, indicated by the green arrow. Microvilli are shown by the blue arrow and the cell-cell junctions by the pink arrow. Treatment with IC_50_ and 2xIC_50_ (Figure 3D,E; image c–f) indicates partial loss of cell membrane integrity (red arrow), absence of microvilli, and disruption of cell junctions (yellow arrow). Figure 3F (images a,b) shows the NC HaCaT cells and the green arrow points to the typical morphology of the lineage, with a fusiform shape. The pink arrow shows the cell junctions and the blue arrow shows the integrated and delimited cell membrane. After the treatments (19 and 38 µM) they seem to be more rounded (white arrows); however, they maintained the intact membrane, and their cellular connections were not altered.

### 3.3. A3K2A3 Reduce Cell Volume in Cervical Cancer Cells

**Figure 4:**
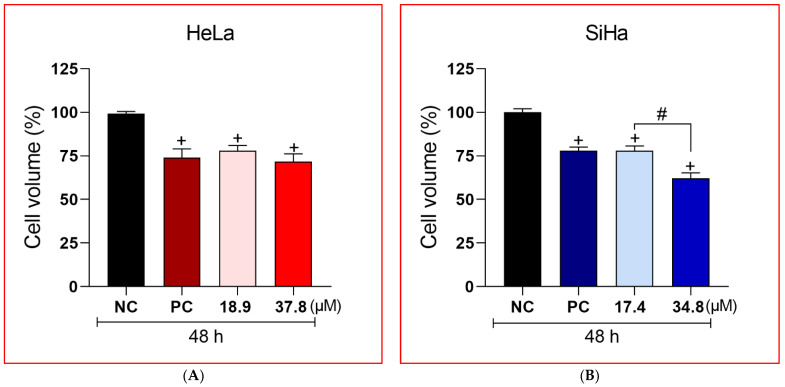
Effects of **A3K2A3** on cervical cancer cell volume. Cells were treated with **A3K2A3** (IC_50_ or 2xIC_50_) for 48 h. The PC was CPT (30 μM). Cell volume was analyzed by flow cytometry. Data represent the mean ± SD of three independent experiments that were performed in triplicate. (**A**) HeLa and (**B**) SiHa; + *p* < 0.05 compared to 48 h NC, # *p* < 0.05 significant difference between treatment concentrations (One-way ANOVA followed by post-Tukey test).

Following the morphological changes visualized by microscopy in both types of treated cancer cells, the determination of cell volume was performed by flow cytometry (Figure 4A,B). HeLa and SiHa cells treated with the IC_50_ showed a reduction in cell volume of 22 and 21%, respectively. Treatments with 2xIC_50_ concentrations ledl to even greater cell volume reductions (28 and 37.5% for HeLa and SiHa, respectively) compared to the NC. CPT was used as a PC and induced a notable decrease in the cell volume for both cell types (26 and 22% for HeLa and SiHa cells, respectively). The treatments for 24 h did not show significant differences in relation to the NC.

### 3.4. A3K2A3 Induces Oxidative Stress in Cells

#### Increased Production of ROS and Reduced Level of Antioxidant Defense in Cervical Cancer Cells

**Figure 5:**
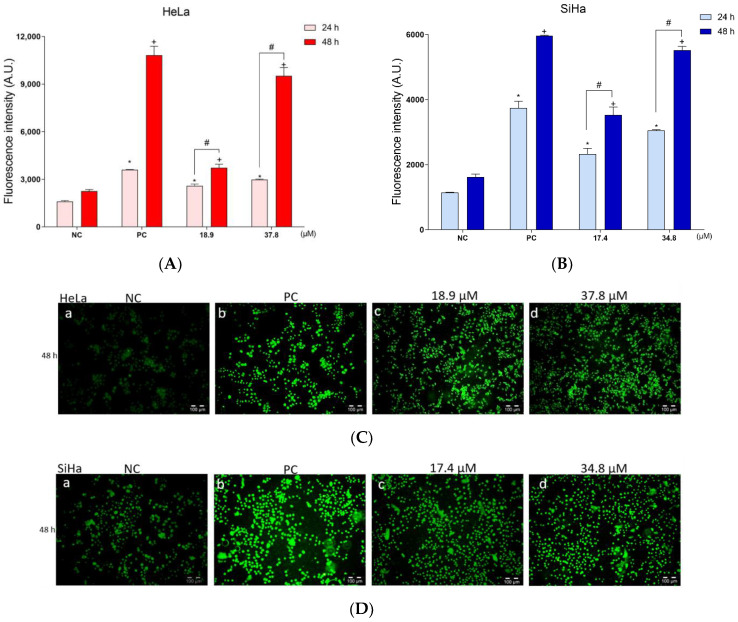

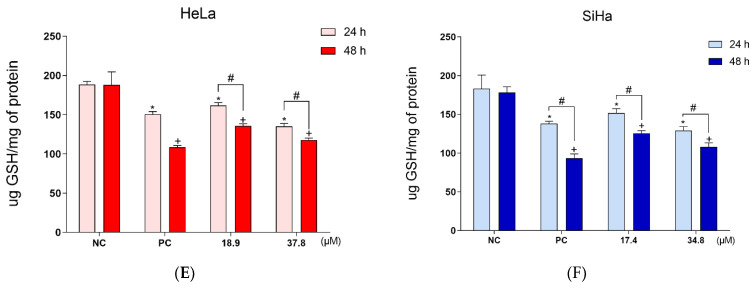
Assessment of oxidative stress. Determination of ROS by the dye H_2_DCFDA in HeLa (**A**,**C**) and SiHa (**B,D**). Assessment of GSH levels by OPA method in HeLa (**E**) and SiHa (**E**) cells. For both experiments, cells were treated with **A3K2A3** (IC_50_ or 2xIC_50_) for 24 and 48 h. The PC was H_2_O_2_ (200 μM). Fluorescence intensity was obtained by spectrofluorimetry. Data represent the mean ± SD of three independent experiments that were performed in triplicate. * *p* < 0.05 compared to 24 h NC, + *p* < 0.05 compared to 48 h NC, # *p* < 0.05 significant difference between 24 and 48 h incubation times (two-way ANOVA followed by Tukey’s post-test). In (**C**,**D**), HeLa and SiHa were photographed using an Olympus^®^ BX51 fluorescence microscope coupled with a UC30 camera (magnification 10×; scale bar: 100 μm), after 48 h treatment.

To investigate possible oxidative damage caused by **A3K2A3** in the cells, the production of total ROS was investigated. HeLa and SiHa cells showed a significant increase in ROS production after 24 h of treatment with IC_50_ (62 and 102%, respectively) and 2xIC_50_ (86 and 166%, respectively). After 48 h treatments, there was a more pronounced increase in ROS levels, around 65 and 117% (IC_50_) and 323 and 240% (2xIC_50_) for HeLa and SiHa, respectively. H_2_O_2_ was used as PC and induced an increase greater than 125 and 225% at 24 h, and 381 and 267% at 48 h for HeLa and SiHa, respectively (Figure 5A,B).

Complementarily, the production of ROS was evaluated by fluorescence microscopy (Figure 5C,D). The NC cells (Figure 5C,D; image a) showed low green fluorescence intensity. However, after treatments for 48 h with **A3K2A3** (both IC_50_ and 2xIC_50_) cells showed an increased green fluorescence (Figure 5C,D; images c,d). Cells pre-incubated with H_2_O_2_ (PC) exhibited intense green fluorescence (Figure 5C,D; image b). Thus, the results demonstrated by fluorescence microscopy are corroborated by the quantifications obtained by spectrofluorimetry.

Considering the increase in ROS production in HeLa and SiHa cells treated with **A3K2A3**, the antioxidant defense system was also analyzed through GSH levels (Figure 5E,F). HeLa and SiHa cells showed a significant reduction in GSH levels after 24 h of treatment with IC_50_ (15 and 18%, respectively) and 2xIC_50_ (19 and 31%, respectively) compared to the NC cells. In treatments for 48 h, there was an even greater decrease in antioxidant defense levels of 29 and 28% (IC_50_) and 39 and 36% (2xIC_50_) for HeLa and SiHa, respectively, compared to the NC cells. H_2_O_2_ was used as a PC and showed a significant decrease of greater than 20 and 24% after 24 h and 42 and 47% after 48 h of treatment, for HeLa and SiHa, respectively. These data demonstrate that the increase in ROS induced by **A3K2A3** led to a reduction in GSH levels, generating oxidative stress in both cell lines.

### 3.5. A3K2A3 Decreases in ΔΨm and Intracellular ATP Levels in Cervical Cancer Cell Lines

**Figure 6:**
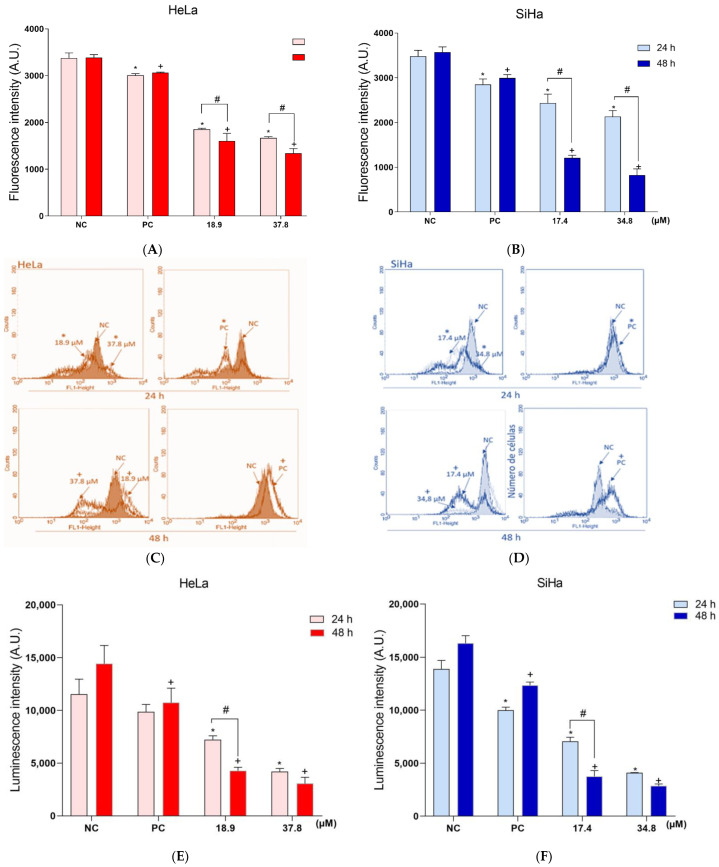
Effects of **A3K2A3** on ΔΨm and intracellular ATP levels. Quantification of ΔΨm by TMRE marker in HeLa (**A**) and SiHa (**B**) cells. Quantification of ΔΨm by Rh 123 marker in HeLa (**C**) and SiHa (**D**) cells. Determination of intracellular ATP levels by Cell Titer-GLO reagent in HeLa (**E**) and SiHa (**F**) cells. For all assays, cells were treated with **A3K2A3** (IC_50_ or 2xIC_50_) for 24 and 48 h. The PCs were CCCP (100 µM) and KCN (1000 µM). Fluorescence intensity was obtained by spectrofluorimetry and flow cytometry. Data represent the mean ± SD of three independent experiments that were performed in triplicate. * *p* < 0.05 compared to 24 h NC, + *p* < 0.05 compared to 48 h NC, # *p* < 0.05 significant difference between 24 and 48 h incubation times (two-way ANOVA followed by Tukey’s post-test).

Considering the oxidative stress generated in HeLa and SiHa treated with **A3K2A3**, the ΔΨm and intracellular ATP levels were analyzed (Figure 6A–F). ΔΨm was first analyzed using the cationic fluorophore TMRE and the two cell lines treated with **A3K2A3** showed a dose-dependent reduction in ΔΨm (Figure 6A,B). HeLa and SiHa cells showed a significant reduction in ΔΨm after 24 h of treatment with IC_50_ (45 and 30%, respectively) and 2xIC_50_ (51% and 39%, respectively). After 48 h treatment, there was an even greater decrease in ΔΨm of 52% and 66% (IC_50_) and 61% and 76% (2xIC_50_) for HeLa and SiHa, respectively, compared to the NC. The mitochondrial membrane uncoupler, CCCP, was used as a PC and induced ΔΨm depolarization greater than 11% and 17% at 24 h and 10% and 16% at 48 h, for HeLa and SiHa, respectively.

In order to confirm the ability of **A3K2A3** to induce ΔΨm depolarization, it was further analyzed using Rh123, and the results were obtained by flow cytometry (Figure 6C,D). The treatment performed on HeLa and SiHa cells with IC_50_ showed a reduction in ΔΨm equal to 24% and 33% after 24 h, and 35% and 74% after 48 h treatments, respectively. Furthermore, in the treatments with 2xIC_50_, the reduction of ΔΨm was around 34 and 51% for 24 h, and 81% and 82% for 48 h for Hela and SiHa, respectively. The PC (CCCP) decreased ΔΨm by more than 5% for both cells and treatment times.

Considering that depolarization of ΔΨm can lead to a reduction in ATP generation, the ATP levels of both cells were measured after exposure to **A3K2A3** using the CellTiter-Glo^®^ reagent (Figure 6E,F). HeLa and SiHa cells showed a significant reduction in intracellular ATP levels after 24 h of treatment with IC_50_ (37% and 49%, respectively) and 2xIC50 (63% and 70%, respectively) compared to the NC cells. In treatments for 48 h, there was an even greater decrease in ATP levels of 70% and 76% (IC_50_) and 78% and 82% (2xIC_50_) for HeLa and SiHa, respectively. The cytochrome oxidase complex inhibitor, KCN, was used as a PC and showed a significant reduction in luminescence intensity by 17% and 28% at 24 h, and 27% and 24% at 48 h, for HeLa and SiHa, respectively.

### 3.6. Effects of A3K2A3 on the Membrane of Cells

#### A3K2A3 Induce LPO and Cell Membrane Damage of Cervical Cancer Cells

**Figure 7:**
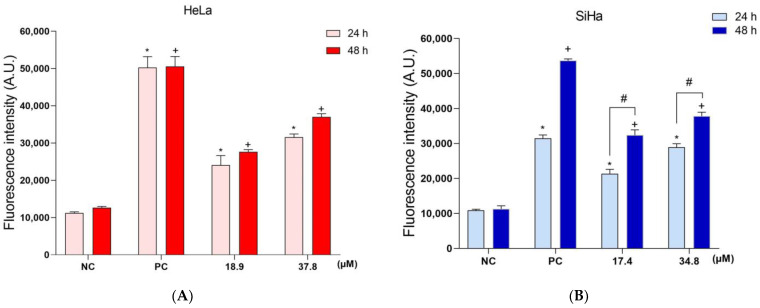

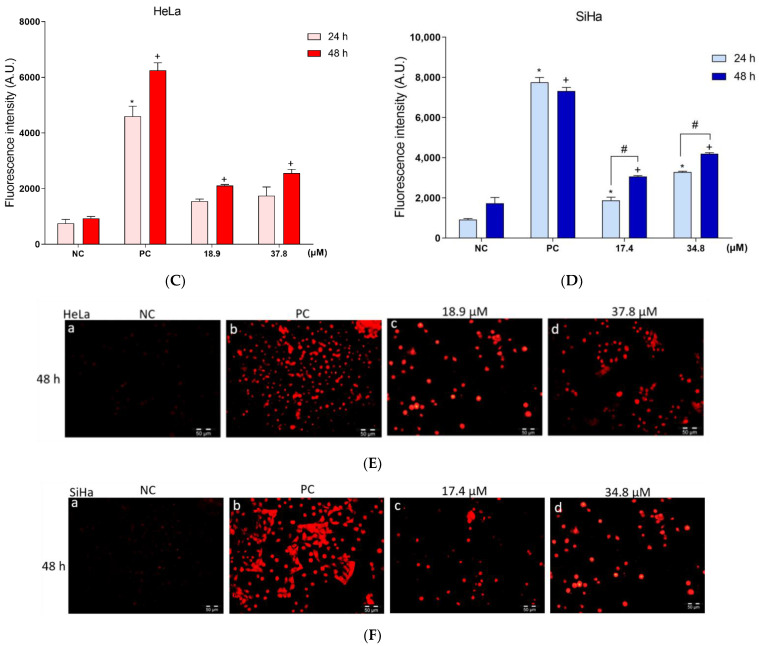
Effects of **A3K2A3** on the membrane of cells. Evaluation of LPO by the DPPP reagent in (**A**) HeLa and (**B**) SiHa. Cell membrane evaluation by PI reagent in HeLa (**C**,**E**) and SiHa (**D**,**F**) cells. For both assays, cells were treated with **A3K2A3** (IC_50_ or 2xIC_50_) for 24 and 48 h. The PCs were H_2_O_2_ (200 µM) and digitonin (80 µM). Fluorescence intensity was performed by spectrofluorimetry. Data represent the mean ± SD of three independent experiments that were performed in triplicate. * *p* < 0.05 compared to the 24 h NC, + *p* < 0.05 compared to the 48 h NC, # *p* < 0.05 significant difference between the incubation times of 24 and 48 h (two-way ANOVA followed by Tukey’s post-test). For images (**E**,**F**), cells were photographed using an Olympus^®^ BX51 fluorescence microscope coupled with a UC30 camera (magnification 20×; scale bar: 50 μm), after 48 h of treatment.

Considering our previous data showing that **A3K2A3** caused oxidative stress and mitochondrial damage in cells, we evaluated whether **A3K2A3** could be triggering molecular and structural changes. First, the LPO was analyzed by means of DPPP labeling (Figure 7A,B). HeLa and SiHa cells showed a significant increase in LPO after 24 h of treatment with IC_50_ (114 and 95%, respectively) and 2xIC_50_ (180% and 165%, respectively) compared to the NC cells. In the treatments for 48 h, there was a more accentuated increase in the LPO of 118% and 186% (IC_50_) and 192% and 234% (2xIC_50_) for HeLa and SiHa, respectively. H_2_O_2_ was used as a PC and induced LPO increases of 346% and 189% at 24 h, and 299% and 375% at 48 for HeLa and SiHa cells, respectively.

As a consequence of LPO, the structural and functional integrity of the cell membrane can be affected, thus it was analyzed by labeling with PI (Figure 7C,D). HeLa and SiHa cells exhibited a significant loss of the cell membrane integrity after 24 h of treatment with IC_50_ (106% and 104%, respectively) and 2xIC_50_ (119% and 257%, respectively) compared to the NC cells. After 48 h, this loss was increased, reaching 126% and 123% (IC_50_) and 174% and 157% (2xIC_50_) for HeLa and SiHa, respectively. Digitonin was used as a PC and induced significant cell membrane damage by increasing the fluorescence by 514% and 745% at 24 h, and 572 and 321% at 48 for HeLa and SiHa cells, respectively.

Additionally, changes in the cell membrane integrity were evaluated by fluorescence microscopy (Figure 7E,F). The NC cells (Figure 7E,F; image a) showed low red fluorescence intensity, which is typically found in cells with an integral membrane, as they do not allow the entry of PI. After treatment for 48 h with **A3K2A3** (IC_50_ or 2xIC_50_), the cells showed an increased red fluorescence (Figure 7E,F; images c,d). Cells pre-incubated with digitonin (PC) showed intense red fluorescence (Figure 7E,F; image b).

### 3.7. Effects of A3K2A3 on the DNA of Cells

#### A3K2A3 Induces Cell Cycle Arrest in G2/M, Cell Chromatin Condensation, and DNA Fragmentation in Cervical Cancer Cells

**Figure 8:**
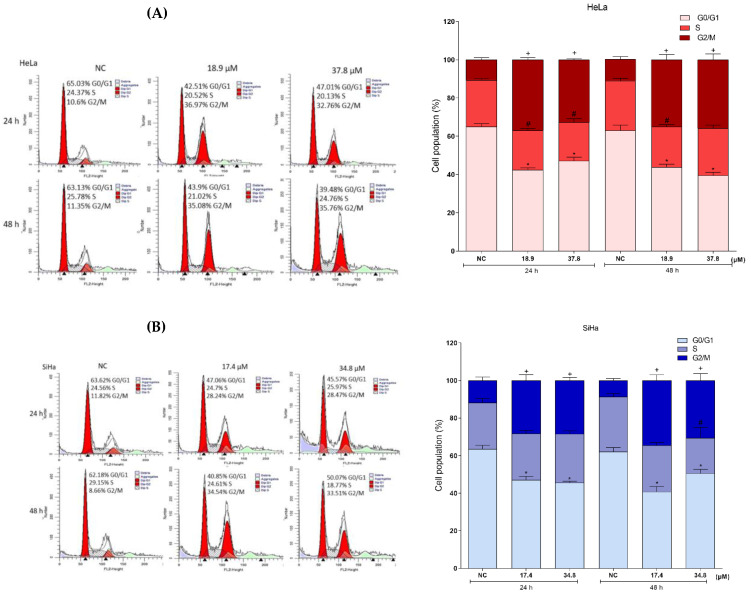

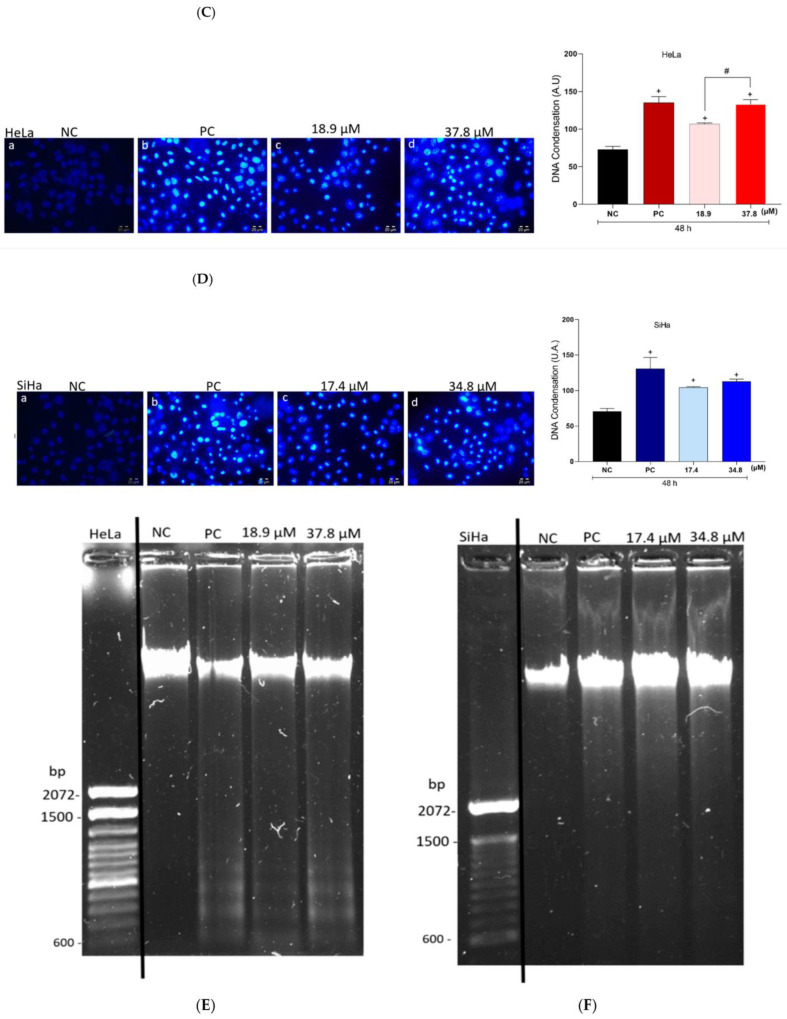
Effects of **A3K2A3** on the DNA of cells. Cell cycle analysis in (**A**) HeLa and (**B**) SiHa. Evaluation of chromatin condensation by Hoechst stain 33342 in (**C**) HeLa and (**D**) SiHa. Evaluation of DNA fragmentation by electrophoresis in 0.75% agarose gel in (**E**) HeLa and (**F**) SiHa. Cells were treated with **A3K2A3** (IC_50_ or 2xIC_50_) for 24 and 48 h for cell cycle analysis and only 48 h for the other assays. The PC was CPT (10 and 30 µM). Quantifications were obtained by flow cytometry (**A** e **B**), spectrofluorimetry (**C**,**D**), and DNA fragmentation was photographed using GelDoc^®^ XR+ Imaging System equipment (**E**,**F**). Data represent the mean ± SD of three independent experiments that were performed in triplicate. (**A**) HeLa; (**B**) SiHa; * *p* < 0.05 compared to G0/G1 phase NC at 24 and 48 h, # *p* < 0.05 compared to S phase NC at 24 and 48 h, + *p* < 0.05 compared to G2/M phase NC at 24 and 48 h (One-way ANOVA followed by Tukey’s post-test). (**C**) HeLa; (**D**) SiHa; + *p* < 0.05 compared to 48 h NC, # *p* < 0.05 significant difference between treatment concentrations (One-way ANOVA followed by Tukey’s post-test). (**E**) HeLa and (**F**) SiHa; The molecular weight marker was the 100 bp DNA Ladder. The experiments were performed in triplicate, reproducing similar results.

Based on the fact that the G1 phase of the cell cycle is responsible for the synthesis of proteins and enzymes necessary for DNA replication, the S phase increases DNA synthesis and G2/M performs the duplication of genetic material and cell division [42]. Damage to the DNA of cells can trigger the arrest of the cell cycle, thus the different phases of the cycle were analyzed by flow cytometry after cell permeabilization and PI labeling (Figure 8A,B). After treatment for 24 h, HeLa cells exhibited reductions in cell populations in the G0/G1 phase by 22% and 18% (IC_50_ and 2xIC_50_, respectively). Concomitantly, we observed increases in cell populations in the G2/M phase of 26% and 22% (IC_50_ and 2xIC_50_, respectively) compared to the NC cells. Treatments for 48 h showed similar results, with reductions of cell population in G0/G1 of 19% and 27% (IC_50_ and 2xIC_50_, respectively), and increases of more than 23% in the G2/M population. SiHa cells after treatments for 24 h showed reductions in the G0/G1 cell populations of more than 15% and cell populations in G2/M increased by 16%. Treatment of cells for 48 h reduced the cell populations in the G0/G1 phase by 21% and 11% (IC_50_ and 2xIC_50_, respectively) and led to cell accumulations of more than 26% in G2/M. Thus, the data showed that treatment with **A3K2A3** led to a notable accumulation of HeLa and SiHa cells in the G2/M phase, suggesting cell cycle arrest in this phase.

Another parameter that reveals DNA damage in cells is chromatin condensation, and this was evaluated by Hoechst staining (Figure 8C,D). NC cells showed intact nuclei and treated cells presented significant nuclear condensation (bright blue color). In the 48 h treatments, the HeLa and SiHa cells showed a marked increase in the fluorescence intensity, indicating chromatin condensation of 47% and 48% (IC_50_), and 82% and 60% (2xIC_50_) for HeLa and SiHa, respectively, in comparison to the NC cells. CPT (10µM) was used as a PC and induced chromatin condensation by 84% and 85% after 48 h of treatment for HeLa and SiHa cells, respectively.

In order to confirm the above results, the effect of **A3K2A3** on cell DNA was also analyzed qualitatively using the agarose gel electrophoresis technique (Figure 8E,F). HeLa and SiHa cells after treatments for 48 h with IC_50_ or 2xIC_50_ showed a strong drag of DNA fragments, indicating intense fragmentation compared to the NC groups, which showed intact DNA bands. The PC, CPT, (30µM) significantly increased cellular DNA fragmentation. Thus, the three assays demonstrated the occurrence of DNA damage in cells induced by **A3K2A3**.

### 3.8. Effects of A3K2A3 on the Possible Type of Death Induced in Cells

#### A3K2A3 Induces Apoptosis in Cervical Cancer Cells

**Figure 9:**
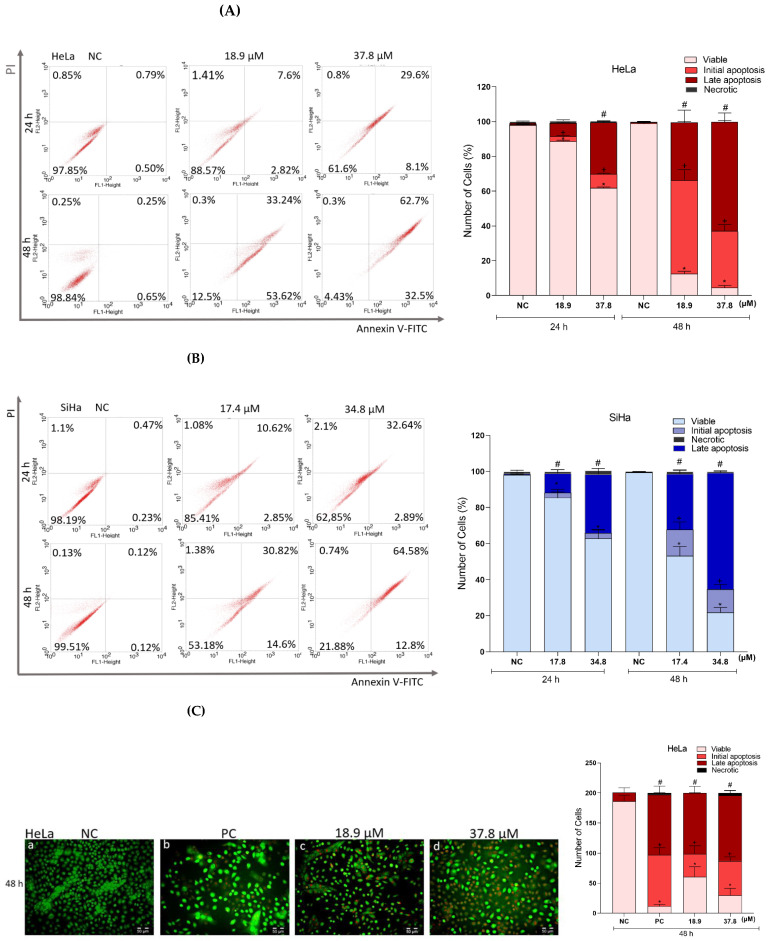

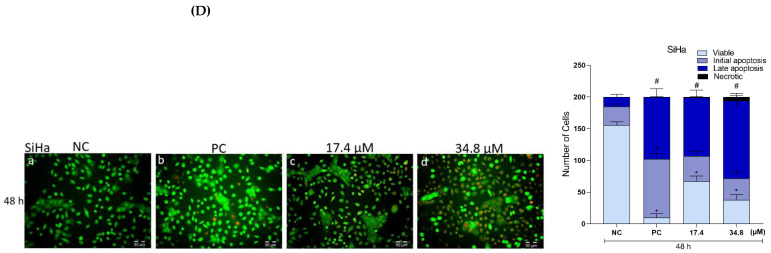
Effects of **A3K2A3** on the possible type of death induced in cells. Annexin/IP tagging (**A**) HeLa and (**B**) SiHa. AO/PI marking (**C**) HeLa and (**D**) SiHa. Cells were treated with **A3K2A3** (IC_50_ or 2xIC_50_) for 24 and 48 h for Annexin/PI labeling analysis and only 48 h for AO/PI marking. The PC was CPT (10 and 30µM). Quantifications were obtained by flow cytometry (**A** e **B**). Cells were visualized using an Olympus BX51 fluorescence microscope (Olympus, Tokyo, Japan), and images captured by a UC30 camera (Olympus, Tokyo, Japan) at (20× magnification; scale bar: 50 μm) followed by cell counts at each stage of apoptosis (**C** e **D**). Data represent the mean ± SD of three independent experiments that were performed in triplicate. (**A**) HeLa; (**B**) SiHa; * *p* < 0.05 compared to viable NC cells at 24 and 48 h, + *p* < 0.05 compared to NC cells in early apoptosis at 24 and 48 h, # *p* < 0.05 compared to NC cells in late apoptosis at 24 and 48 h (one-way ANOVA followed by Tukey’s post-test). (**C**) HeLa; (**D**) SiHa; * *p* < 0.05 compared to viable NC cells, + *p* < 0.05 compared to early apoptotic NC cells, # *p* < 0.05 compared to late apoptotic NC cells.

To determine whether the cell death mechanism triggered by **A3K2A3** involves apoptosis, we evaluated the externalization of phosphatidylserine (Figure 9A,B). In HeLa cells, the percentage of cells in initial apoptosis increased by 2.3% and 7.6% after 24 h of treatment, while for late apoptosis it increased by 6.81% and 28.88% (IC_50_ and 2xIC_50_, respectively). In treatments for 48 h, the results were similar, showing an increase of 52.9% and 31.9% (IC_50_ and 2xIC_50_, respectively) in the cell population in early apoptosis and 32.9% and 62.4% in late apoptosis, respectively, with a reduction of more than 86% of viable cells. The treatment of SiHa cells for 24 h caused an increase in the percentage of cells in initial apoptosis of 2.6% and 2.7%, whereas late apoptosis increased by 10% and 32% (IC_50_ and 2xIC_50_, respectively). In treatments performed for 48 h, increases of 14% and 12% in the cell populations in early apoptosis and 30% and 64% in late apoptosis were observed (IC_50_ and 2xIC_50_, respectively), and a reduction in the percentage of viable cells greater than 46% was observed. The percentage of cells in necrosis was not significant in any of the treatments or times.

In order to confirm the results of the previous experiment, the quantification of apoptotic cells was also performed by means of AO/PI double labeling (Figure 9C,D). For HeLa cells, treatment for 48 h showed an increase of more than 1000% of cells in early apoptosis, and for late apoptosis it increased by 605% and 660% (IC_50_ and 2xIC_50_, respectively) and, at the same time, we observed reductions in the percentage of viable cells of 67% and 84% (IC_50_ and 2xIC_50_, respectively). In SiHa cells, treatments for 48 h showed an increase in cells in initial apoptosis of 36% and 16%, and for late apoptosis, it increased by 517% and 690% (IC_50_ and 2xIC_50_, respectively). There was also a reduction in viable cells (57% and 76% for IC_50_ and 2xIC_50_, respectively). The percentage of necrotic cells did not present significant results in any of the treatments. The PC, CPT (10 μM) showed an increase in the percentage of cells in early and late apoptosis of more than 40% for both cell types.

**Figure 10:**
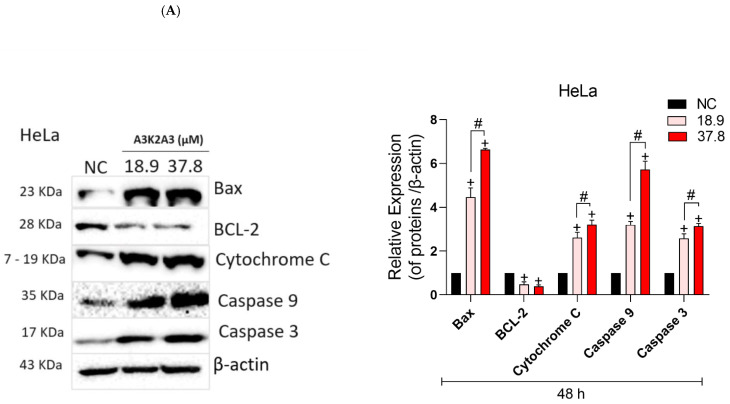

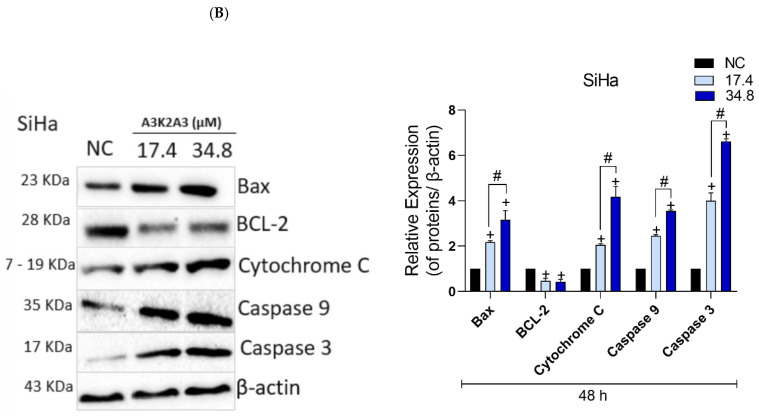
Effects of **A3K2A3** on the expression of apoptosis-related proteins. Western Blotting (**A**) HeLa and (**B**) SiHa. Cells were treated with **A3K2A3** (IC_50_ or 2xIC_50_) for 48 h. The antigen-antibody complex was detected by chemiluminescence employing ECL detection reagent and analyzed using ChemiDoc^®^ XRS+ Imaging System and the protein levels were determined by normalization with β-actin. Data represent the mean ± SD of three independent experiments that were performed in triplicate. (**A**) HeLa; (**B**) SiHa; + *p* < 0.05 compared to 48 h NC, # *p* < 0.05, a significant difference between **A3K2A3** concentrations (one-way ANOVA followed by Tukey’s post-test).

To further explore the underlying mechanism of **A3K2A3**-induced apoptosis in HeLa and SiHa cells, we detected the possible involvement of apoptosis-related proteins by Western blotting analysis. The results showed that treatment with **A3K2A3** at concentrations of IC_50_ and 2xIC_50_ increased the expression levels of the pro-apoptotic protein Bax in a concentration-dependent manner by 340% and 110% (IC_50_) and 570% and 210% (2xIC_50_) for HeLa and SiHa, respectively (Figure 10). Meanwhile, the expression level of the anti-apoptotic protein Bcl-2 was downregulated, with its expression decreased by 52% and 54% (IC_50_) and 60% and 58% (2xIC_50_) for HeLa and SiHa respectively (Figure 10).

Our results also showed that **A3K2A3** significantly increased cytoplasmic cytochrome C protein levels by 160% and 100% (IC_50_) and 220% and 310% (2xIC_50_) for HeLa and SiHa respectively (Figure 10). In addition, it was possible to observe that the expression of the caspase 9 protein was significantly increased in both cells treated with **A3K2A3**: 210% and 140% (IC_50_) and 470% and 250% (2xIC_50_) for HeLa and SiHa respectively (Figure 10). Finally, it was observed that caspase 3 showed an increase in its expression of 150% and 300% (IC_50_) and 210% and 550% (2xIC_50_) for HeLa and SiHa respectively (Figure 10). Thus, the results demonstrated by three different assays corroborated the possible induction of cell death by apoptosis triggered by **A3K2A3** in cervical cancer cells.

### 3.9. Effects of A3K2A3 on Cell Survival

#### A3K2A3 Reduces Colony Formation of Cervical Cancer Cells

**Figure 11:**
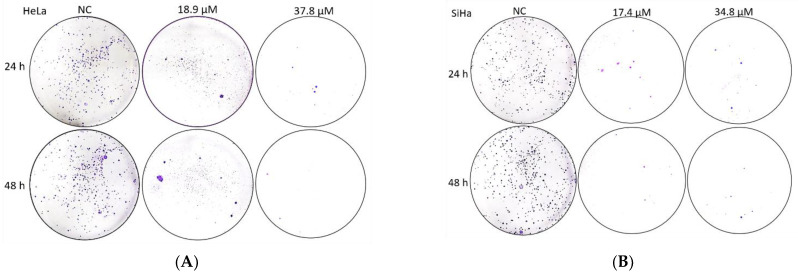

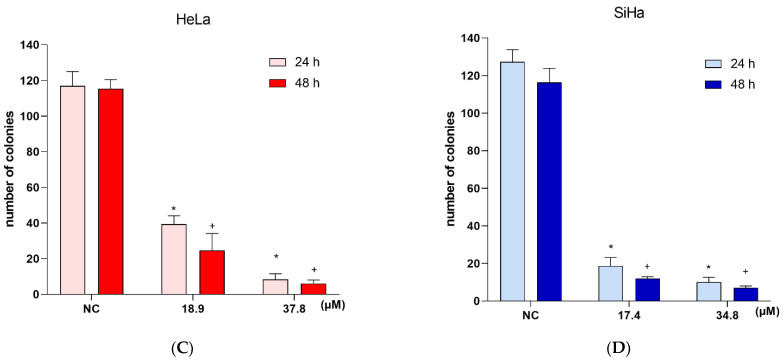
Effects of **A3K2A3** on cell survival. Colonies of (**A**) HeLa and (**B**) SiHa stained with Giemsa in 6-well plates. Quantification of the number of colonies in relation to the NC (**C**) HeLa and (**D**) SiHa. Cells were treated with **A3K2A3** (IC_50_ or 2xIC_50_) for 24 and 48 h. Colonies were counted and plotted on graphs showing the average number of colonies for the NC versus treated cells. A cluster of cells was considered a colony when it contained more than 50 cells. The bars represent the mean ± SD of three independent experiments that were performed in triplicate. (**C**,**D**) * *p* < 0.05 compared to 24 h NC, + *p* < 0.05 compared to 48 h NC (two-way ANOVA followed by Tukey’s post-test).

This assay demonstrated that treatment of HeLa and SiHa cells with **A3K2A3** was able to prevent cell adhesion, which can be observed by the inhibition of colony formation (Figure 11A,B). Both cell types showed a reduction in colony formation of greater than 66% after 24 h of treatment at both concentrations. In treatments for 48 h, there were reductions in the number of colonies of 78% and 89% for IC_50_ and 94% and 93% for 2xIC_50_ for HeLa and SiHa cells, respectively.

### 3.10. Effects of A3K2A3 on Cell Migration

#### A3K2A3 Inhibited Cell Migration and Decreases the Expression of MMP-9 in Cervical Cancer Cells

**Figure 12:**
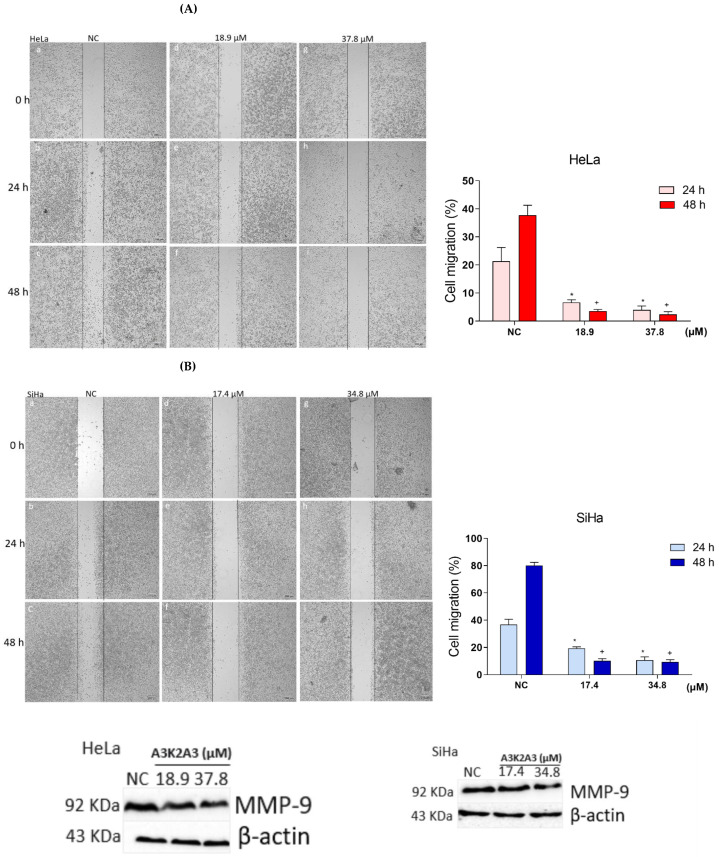

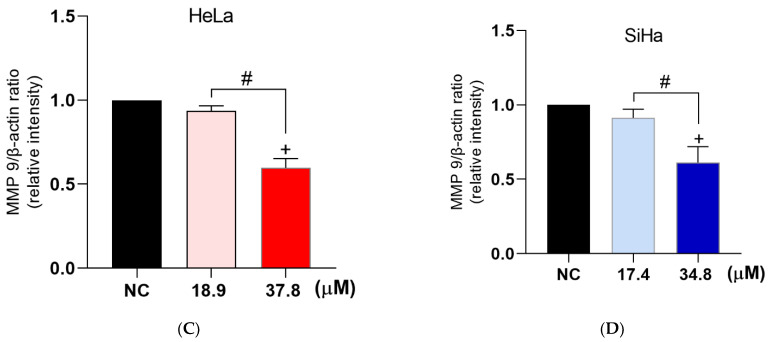
Effects of **A3K2A3** on cell metastasis. Analysis of cell migration (**A**) HeLa and (**B**) SiHa. Evaluation of MMP-9 expression (**C**) HeLa and (**D**) SiHa. Cells were treated with **A3K2A3** (IC_50_ or 2xIC_50_) for 24 and 48 h for analysis of cell migration and only 48 h for evaluation of MMP-9 expression. The photos were taken using an Olympus CKX41 inverted phase contrast microscope (4× magnification; scale bar: 200 μm) and the percentage of cell growth in the wound region was calculated using ImageJ software (**A**,**B**). The antigen-antibody complex was detected by chemiluminescence employing ECL detection reagent and analyzed using ChemiDoc^®^ XRS+ Imaging System, and the protein levels were determined by normalization with β-actin (**C**,**D**). (**A**) HeLa and (**B**) SiHa; NC (a, b, c), IC_50_ at 0, 24, and 48 h (d, e, f) and 2xIC_50_ at 0, 24, and 48 h (g, h, i). The data represent the percentage of wound closure ± SD of three independent experiments that were performed in triplicate. * *p* < 0.05 compared to 24 h NC, + *p* < 0.05 compared to 48 h NC (two-way ANOVA followed by Tukey’s post-test). (**C**) HeLa and (**D**) SiHa; data represent the mean ± SD of three independent experiments that were performed in triplicate. + *p* < 0.05 compared to 48 h NC, # *p* < 0.05 significant difference between **A3K2A3** concentrations (one-way ANOVA followed by Tukey’s post-test).

Cell migration was analyzed using the wound healing assay as shown in Figure 12A,B. For HeLa cells, the percentage of cell migration in the NC was 22 and 37% at 24 and 48 h, respectively (Figure 12A; images a–c). Regarding SiHa cells, the percentage of cell migration in the NC was 36% and 70% at 24 and 48 h, respectively (Figure 12B; images a–c). Treatment with **A3K2A3** inhibited cell migration for both cell types. It was observed that migration was less than 7% for HeLa (Figure 12A; images d–i) and less than 18% for SiHa cells (Figure 12B; images d–i) after 24 and 48 h of treatment, respectively.

In order to clarify one of the possible mechanisms by which **A3K2A3** reduces cervical cancer cell migration, the levels of MMP-9 expression were investigated (Figure 12C,D). For both cell types, treatment with IC_50_ did not significantly suppress MMP-9 expression; however, treatments with 2xIC_50_ led to remarkable reductions in MMP-9 expression of 41% and 38% for HeLa and SiHa, respectively, compared to the NC. Our results suggest that the negative regulation of MMP-9 expression may be contributing to the decrease in cell migration, but probably other mechanisms are involved in this process.

## 4. Discussion

Although cervical cancer screening and prevention measures help to reduce cases of the disease in developed countries, in developing regions this cancer remains one of the main causes of death for women [9,43,44], and considering the limitations of the current treatments available, many studies are focusing on the discovery of new therapeutic approaches that can increase the specificity and efficacy of anticancer agents to reduce adverse effects [45]. Studies have demonstrated the different therapeutic properties of DBAs and their derivatives [18,19,20,21,22,23,24,46], however, there are no reports yet of the cellular mechanism associated with anticancer activity and the biological function of the synthetic DBA **A3K2A3** in cervical cancer.

In this study, we investigated its cytotoxic activity and mechanism of action in HeLa and SiHa cervical cancer cells. **A3K2A3** dose-dependently reduced the viability of both cell lines, proving the in vitro antitumoral activity of this compound. Similar data on the cytotoxic activity of DBAs have already been observed in oral cancer cells [20]. Shi [12] also reported inhibition of ovarian cancer cell growth after treatment with curcumin, a DBA analog. Additionally, **A3K2A3** showed a more selective cytotoxic effect for cervical cancer cells compared to HaCaT. SI values > 2 indicate a likely value as an anticancer agent [47,48].

We also evaluated the cytotoxic effect of **A3K2A3** by microscopy and observed that it induced pronounced morphological changes in the two tested cervical cancer cells, in addition to changes in the cell membrane. In contrast, no significant morphological changes were observed in HaCaT cells exposed to concentrations greater than the IC_50_ and 2xIC_50_ of cancer cells for the same period of time. The reduction in cell volume was confirmed by data obtained from flow cytometry. A decrease in cell volume induces morphological changes, also constituting a characteristic of cell death by apoptosis [49]. Other studies with cancer cells showed similar changes to those found in our work and suggested a process of cell death by apoptosis [12,50].

Considering these morphological changes, we further investigated whether exposure to **A3K2A3** induces oxidative stress. Oxidative stress results from the imbalance between the generation of ROS and the activity of the antioxidant system [28]. ROS is responsible for regulating several cancer-related signaling pathways, in addition to being an important regulatory signal of apoptosis in tumor cells [51]. The generation of ROS in cells exists in balance with a variety of antioxidant defenses [52]. One of the main mechanisms of resistance in tumor cells (to multiple substances, to radiation, and chemotherapeutics) when the production of ROS is increased is through the increase of antioxidant production. Several antioxidant pathways that inhibit ROS are regulated during tumor initiation and progression [53]. The antioxidant system comprises a series of enzymes that play a relevant role in the detoxification of hydroperoxides [53]. Therefore, this relationship justifies many studies, explaining cancer cell death is mediated by an increase in ROS production associated with a reduction in the levels of antioxidant defenses [54,55].

Our results showed that **A3K2A3** led to an increase in the fluorescence intensity of DCF-DA in HeLa and SiHa cells, indicating a high production of ROS, which intensified after 48 h of treatment for both cell types. This excessive production possibly caused a redox imbalance leading to oxidative damage, thus exceeding the repair capacity of the antioxidant system. We demonstrated that both cells had a depletion of GSH levels, characterizing a state of oxidative stress in these cells. These results reinforce the theory that apoptosis may be the cell death pathway induced by **A3K2A3,** and that it is possibly a result of oxidative stress. Other tumor cell studies have also suggested that the accumulation of ROS and the reduction of GSH levels resulted in a redox imbalance, leading to the oxidation of biomolecules vital for cellular functions, and activation of apoptotic cell death pathways [11,56,57,58].

Mitochondria are a major source of intracellular ROS, and a sharp increase in their production can result in dysfunctional mitochondria and consequent reduction in ATP levels [52,59]. Depolarization of the Δψm is one of the main factors that contributes to the induction of apoptosis in tumor cells [50,60]. In this study, depolarization of Δψm was observed, in addition to a reduction in intracellular ATP levels of HeLa and SiHa cells. Studies concerning cervical cancer cells have also demonstrated depolarization of the mitochondrial membrane in which mitochondrial damage was associated with the induction of apoptosis via an intrinsic pathway [50,58].

Oxidative stress can damage cellular structures, including lipids, nucleic acids, and proteins, thus leading to DNA damage [11,48,52,61,62]. LPO affects the structural and functional integrity of cell membranes, inducing changes in their permeability, and is associated with the oxidative degradation of lipids, which are susceptible to free radicals, leading to cell membrane impairment [63]. When oxidative degradation of lipids occurs, the permeability of the membrane increases and interrupting a series of vital biochemical processes within it and eventually leading to its death [64].

In this study, **A3K2A3** induced significant LPO in the two cervical cancer cell lines. Disturbances in cell membrane integrity were also demonstrated by PI labeling. These results are consistent with the data that had already been observed through the images obtained by scanning electron microscopy and are also reinforced by the images obtained in AO/PI double labeling. Thus, these findings suggest that the alterations observed in the cell membrane can collaborate in the induction of cell death.

It is known that high levels of ROS can induce cellular DNA damage [29], and this damage can lead to cell cycle dysregulation followed by cell death by apoptosis [65]. Checkpoints G0/G1, S, and G2/M are crucial steps that ensure cells can progress to each phase of the cycle without any defects [66]. The presence of DNA damage can lead to failures in these checkpoints, preventing DNA replication and mitosis [67], which can trigger apoptosis in order to eliminate abnormal cells. DNA damage, resulting from dysregulation during the cell cycle or other factors such as an increase in ROS, activates apoptotic mechanisms, which allow cellular transformation to be avoided [65].

Cell cycle arrest in the G2/M phase is one of the most prominent checkpoints of many anticancer agents that can reduce cell proliferation and then induce apoptosis by inhibiting the segregation of damaged chromosomes during mitosis [67]. Our work revealed that exposure of HeLa and SiHa cells to **A3K2A3** resulted in cell cycle arrest in the G2/M phase, accompanied by a decrease in cells in G0/G1, which suggests apoptosis of these cells in addition to blocking cell division in the mitotic phase. This hypothesis is supported by other studies that also reported that chalcones derivatives significantly blocked cell proliferation in the G2/M phase, helping to induce apoptosis [11,68]. Furthermore, it was consistent with Li et al. [69], in which HeLa cells treated with a lipophilic grape seed Proanthocyanidin compound (LGSP) induced an increase in ROS production and led the cells to apoptosis, in addition to generating cell cycle blockade in G2/ M.

Considering the hypothesis that DNA damage in cells leads to cell cycle interruption in the G2/M phase, we further characterized this damage by analyzing chromatin condensation and DNA fragmentation. It was possible to observe that the proportion of cells with condensed chromatin and fragmentation of DNA were significantly increased after treatment with **A3K2A3**, corroborating the apoptotic process in the two cervical cancer cell lines. Chromatin condensation and DNA degradation are considered essential steps in the characterization of apoptosis [65]. Supporting our results, DNA fragmentation and cellular chromatin condensation have previously been observed in cervical cancer cells, contributing to the confirmation of an apoptotic process [40,70].

Collectively, our data provide evidence that **A3K2A3** may be inducing apoptosis in both cell types. Thus, to further confirm the cell death mechanism, Annexin-V/IP double labeling assays were performed. Our results confirmed that **A3K2A3** is inducing late apoptosis in HeLa and SiHa cells, and it was more intense after treatment for 48 h. Other studies with DBAs have indicated the induction of apoptosis in several cancer cell lines [19,20,21], reinforcing our findings.

In addition, the labeling of cells with AO/PI also corroborated the classification of the type of cell death, as an increase in the percentage of cells in late apoptosis (represented by the double labeling of AO/PI) was observed. Our results are in agreement with reports that cervical cancer cells treated with a fraction of Stryphnodendron adstringens revealed, by transmission electron microscopy, an intense disruption in the cell membrane, and the authors suggested a feature of late apoptosis, confirming this hypothesis by double labeling with AO /PI [40].

Finally, aiming to further explore the mechanism of apoptosis induced in the cells by **A3K2A3**, we evaluated the expression of some key proteins in the regulation of this cell death. Mitochondria play an important role in the apoptotic pathway of tumor cells [71]. When mitochondrial depolarization occurs, pro-apoptotic proteins are highly expressed and can result in the formation of pores in the outer membrane of mitochondria, which is controlled by the BCL-2 family, mainly by the pro-apoptotic factors Bax and the anti-apoptotic factors BCL-2 [59].

The balanced relationship between pro-apoptotic and anti-apoptotic proteins regulates the process of apoptosis [72]. An increase in Bax protein levels leads to the formation of pores in the mitochondrial membrane, resulting in the release of pro-apoptotic factors into the cytosol. The BCL-2 protein prevents this release in order to protect the cell from apoptosis [73] but, due to mitochondrial depolarization caused by oxidative stress, its expression is reduced, leading to the release of cytochrome C from the mitochondria to the cytoplasm, which induces the formation of apoptotic bodies and the activation of caspase family proteins, resulting in irreversible cell death [72].

Our results showed that the expression of Bax was upregulated while the expression of Bcl-2 was downregulated, leading to the release of cytochrome C to the cytosol, thus increasing its expression which consequently led to the activation of Caspase family proteins (Caspase- 9 and Caspase-3). These findings reinforce the hypothesis that cell exposure to **A3K2A3** is probably inducing a process of cell death by apoptosis via mitochondrial damage mediated by the overproduction of ROS, with the consequent opening of transport pores, which promotes the release of pro-apoptotic agents activating the apoptosis cascade. Another study revealed that 1’-acetoxyeugenol acetate (AEA) induced apoptosis in human ovarian cancer cells, activating the intrinsic pathway by inducing an increase in intracellular ROS levels, and this induction was determined by an increase in DCF-DA fluorescence intensity [74].

After the cells were exposed to **A3K2A3** and subsequently induced to grow again, it was possible to assess the cell survival capacity through colony formation. We detected that **A3K2A3** was able to prevent the formation of colonies, and this result is possibly related to the fact that the compound induces DNA damage and blocks cells from mitosis in the G2/M phase, preventing its multiplication. Colony formation occurs from a single cell and, therefore, when there is no colony formation, it can be indicated that the treatment induces reproductive cell death as a result of damage to the chromosomes and can lead to apoptosis [41]. Prakobwong [13,14] reported that cancer cells exposed to curcumin led to significant suppression of colony formation capacity, corroborating our results.

The possible potential of **A3K2A3** to prevent cell migration was investigated by evaluating wound healing and the expression of the MMP-9 protein. MMP-9 is a protein known to play a key role in cancer progression and metastasis, including cancer cell invasion, migration, and angiogenesis [5]. It was shown that **A3K2A3** markedly suppressed cell migration in addition to downregulating MMP-9 expression. Our results were consistent with those found by Huang [50], who reported some synthetic compounds inhibited HeLa cell proliferation and migration. Another study, also in cervical cancer cells, demonstrated the suppression of viability, motility, and invasion by decreasing the expression of MMP-9 [5].

In general, our results showed that the action of **A3K2A3** was more intense as the concentration and treatment time increased, a fact that was observed for both cell types. In addition, the results found were similar for both cervical cancer cell lines, which was expected, considering that the cells differ only in the type of HPV that modified them. Our data is supported by other studies that observed similar changes in HeLa and SiHa cells [10,40,56,67,75].

In summary, all these morphological and biochemical changes that were induced by **A3K2A3** in the present study probably caused cumulative damage in the cells that are irreconcilable with cell survival. Considering the promising results found in the study by the single agent **A3K2A3** another important approach in this work would be drug combination. Combined therapy has been a much-studied alternative for clinical application, seeking to achieve a synergistic effect and reduce toxicity, due to the decrease in the effective dose, leading to a possible increase in the effectiveness of the treatment [76,77]. The effects of interaction between target drugs or standard chemotherapy drugs and cytotoxic agents are little investigated [77].

It is worth mentioning that, despite all the in vitro interesting biological properties of **A3K2A3** demonstrated in this work, there is no data on the interaction of this compound and standard chemotherapeutics for the treatment of cervical cancer, in previous literature. Additional studies such as combined therapy are necessary for an attempt that this association presents synergistic effects, enhancing the chemotherapeutic effect, allowing doses reduction of the drugs, as well as toxicity. In addition, an in vivo evaluation model needs to be carried out to evaluate the effective inhibition of tumor growth, in order to provide more data for the consideration of **A3K2A3** as a potential candidate for cervical cancer therapy.

## 5. Conclusions

**Figure 13:**
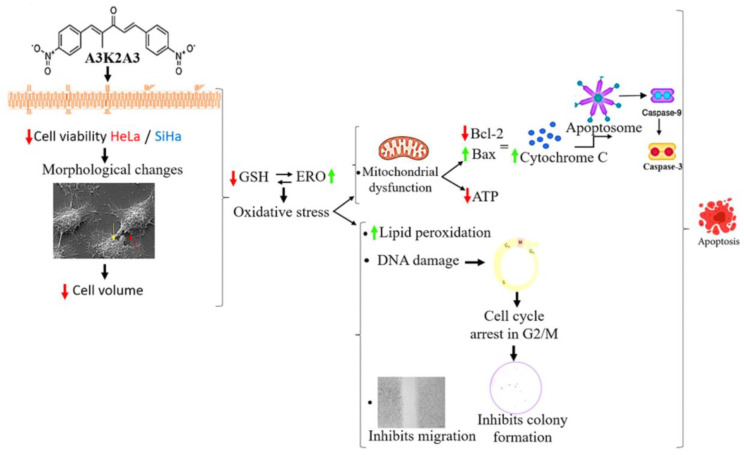
Mechanism of action of dibenzylideneacetone (**A3K2A3**) on cervical cancer cells.

In conclusion, our results showed that **A3K2A3** has a dose-dependent cytotoxic effect on HeLa and SiHa cells and was also observed to be more selective against cancer cells in relation to HaCaT cells. **A3K2A3** further induced redox imbalance in both cells, leading to mitochondrial impairment and low levels of intracellular ATP. In addition, damage to the cell membrane and DNA were demonstrated. Together, these changes possibly triggered a process of apoptosis via mitochondria, confirmed by the positive regulation of apoptotic proteins in both cell lines. **A3K2A3** also showed potential in preventing cell migration and multiplication by blocking cell survival (as shown in Figure 13). These findings indicate **A3K2A3** as a potential candidate in the development of a therapeutic agent for cervical cancer treatment and support future preclinical and clinical studies to validate its antitumor effects.

## Figures and Tables

**Table 1:** IC_50_ of HeLa, SiHa, and HaCaT cell lines, and the SI after 48 h of treatment.

A3K2A3	IC_50_ (µM)	SI
HeLa	18.9 ± ^1^ 2.68	2.54
SiHa HaCaT	17.4 ± 2.89 48.06 ± 2.92	2.75

^1^ ± SD standard deviation.

## Data Availability

The data presented in this study are available in this manuscript.
